# The Subtype Selectivity in Search of Potent Hypotensive Agents among 5,5-Dimethylhydantoin Derived α_1_-Adrenoceptors Antagonists

**DOI:** 10.3390/ijms242316609

**Published:** 2023-11-22

**Authors:** Aneta Kaczor, Joanna Knutelska, Katarzyna Kucwaj-Brysz, Małgorzata Zygmunt, Ewa Żesławska, Agata Siwek, Marek Bednarski, Sabina Podlewska, Magdalena Jastrzębska-Więsek, Wojciech Nitek, Jacek Sapa, Jadwiga Handzlik

**Affiliations:** 1 Department of Technology and Biotechnology of Drugs, Medical College, Jagiellonian University, Medyczna 9, 30-688 Krakow, Poland; aneta.kaczor@uj.edu.pl (A.K.); katarzyna.kucwaj@uj.edu.pl (K.K.-B.); 2 Department of Pharmacodynamics, Medical College, Jagiellonian University, Medyczna 9, 30-688 Krakow, Poland; joanna.1.knutelska@uj.edu.pl (J.K.); malgorzata.zygmunt@uj.edu.pl (M.Z.); marek.bednarski@uj.edu.pl (M.B.); jacek.sapa@uj.edu.pl (J.S.); 3 Institute of Biology and Earth Sciences, University of the National Education Commision, Podchorążych 2, 30-084 Krakow, Poland; ewa.zeslawska@up.krakow.pl; 4 Department of Pharmacobiology, Medical College, Jagiellonian University, Medyczna 9, 30-688 Krakow, Poland; agat.siwek@uj.edu.pl; 5 Maj Institute of Pharmacology, Polish Academy of Sciences, Department of Medicinal Chemistry, Smętna 12, 31-343 Krakow, Poland; smusz@if-pan.krakow; 6 Department of Clinical Pharmacy, Medical College, Jagiellonian University, Medyczna 9, 30-688 Krakow, Poland; m.jastrzebska-wiesek@uj.edu.pl; 7 Faculty of Chemistry, Jagiellonian University, Gronostajowa 2, 30-387 Krakow, Poland; wojciech.nitek@uj.edu.pl

**Keywords:** α_1_-adrenoceptor, 5,5-dimethylhydantoin, crystallography, molecular modelling, α_1A_, α_1B_

## Abstract

In order to find new hypotensive drugs possessing higher activity and better selectivity, a new series of fifteen 5,5-dimethylhydantoin derivatives (**1**–**15**) was designed. Three-step syntheses, consisting of N-alkylations using standard procedures as well as microwaves, were carried out. Crystal structures were determined for compounds **7**–**9**. All of the synthesized 5,5-dimethylhydantoins were tested for their affinity to α_1_-adrenergic receptors (α_1_-AR) using both in vitro and in silico methods. Most of them displayed higher affinity (Ki < 127.9 nM) to α_1_-adrenoceptor than urapidil in radioligand binding assay. Docking to two subtypes of adrenergic receptors, α_1A_ and α_1B_, was conducted. Selected compounds were tested for their activity towards two α_1_-AR subtypes. All of them showed intrinsic antagonistic activity. Moreover, for two compounds (**1** and **5**), which possess o-methoxyphenylpiperazine fragments, strong activity (IC_50_ < 100 nM) was observed. Some representatives (**3** and **5**), which contain alkyl linker, proved selectivity towards α_1A_-AR, while two compounds with 2-hydroxypropyl linker (**11** and **13**) to α_1B_-AR. Finally, hypotensive activity was examined in rats. The most active compound (**5**) proved not only a lower effective dose than urapidil but also a stronger effect than prazosin.

## 1. Introduction

The α_1_-adrenoceptors (α_1_-ARs) belong to the G protein-coupled receptor (GPCR) and play a fundamental role in the physiology of the sympathetic nervous system [1]. They are activated by the endogenous catecholamines, norepinephrine and epinephrine and mediate various physiological effects, such as vascular and urogenital smooth muscle contraction [2]. Pharmacologically, α_1_-ARs are an important cellular target for the treatment of several pathologies [3], e.g., hypertension [4,5,6], benign prostatic hyperplasia (BPH) [7], and lower urinary tract symptoms (LUTS) [8,9]. Nowadays, three subtypes of α_1_-adrenoceptors, namely α_1A_, α_1B_, and α_1D_, have been identified based on molecular cloning techniques and pharmacological studies [1]. All three α_1_-AR subtypes are present in blood vessels [10], whereas the α_1B_ subtype contributes to vasoconstriction and blood pressure control in humans [11,12,13]. Interestingly, the expression of vascular α_1_-AR subtypes changes with age. In the mammary artery, α_1B_-AR expression increases 2-fold in patients aged 65 years or older compared to patients younger than 55 years. Consequently, the α_1B_-AR is the predominant subtype in the arteries in older patients (≥65 years) [10]. Data from human studies indicate that α_1B_-AR predominates in epicardial coronary artery endothelial cells (90–95% of total α_1_-AR mRNA) [14]. Recently, a molecular study confirmed a statistically significant increase in the expression of α_1B_-AR in human peripheral mononuclear cells from hypertension patients. The increase in α_1B_-AR mRNA levels correlates with the increase in systolic blood pressure and plasmatic homocysteine levels [15]. Thus, α_1_-AR antagonists are attractive therapeutic targets for the treatment of hypertension. Blockade of α_1_-ARs reduces vascular resistance and venous capacitance [16], consequently decreasing peripheral resistance and lowering blood pressure [6]. It is also well established that α_1_-adrenoceptor antagonists have beneficial effects on lipids profile and glucose metabolism [17,18,19,20,21,22,23,24,25,26,27]. Recent clinical studies have shown that therapy with α_1_-AR blockers is associated with a lower rate of readmission for heart failure and mortality [28].

The α_1A_- and α_1B_-ARs are present in the human myocardium and do not appear to have an important role in the regulation of heart function under normal physiological conditions but increase importance under pathological conditions [29]. During cardiac ischemia and post-ischemic reperfusion, α_1_-adrenergic stimulation may play a significant role in the generation of arrhythmias because myocardial ischemia causes an increase in α_1_-ARs density and intracellular Ca^2+^ overload [30,31,32,33,34]. The increase in intracellular Ca^2+^ signaling, in response to α_1_-adrenergic stimulation, is mediated by the α_1A_-AR [35]. Data from animal studies demonstrate that the α_1A_-AR antagonists have antiarrhythmic effects [36]. Therefore, this subtype is an interesting target for the treatment of arrhythmias.

**Figure 1:**
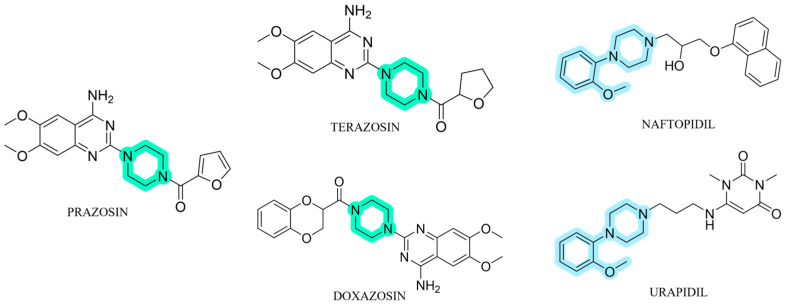
The structures of already approved α_1_-adrenoceptors (α_1_-ARs) antagonists.

In this context, the search for antagonists of α_1_-ARs is an important issue in the discovery and development of new therapeutic agents. Prazosin, which was the first marketed antihypertensive drug with α-adrenolytic activity and two further pharmaceuticals in this class, terazosin and doxazosin [4,37,38], contain a piperazine-1,4-diyl moiety (Figure 1) [39,40]. Pharmacological studies of α_1_-ARs antagonists demonstrated that an o-methoxyphenylpiperazine moiety plays a significant role in the affinity to this subtype [39,40,41,42], and this structural fragment is also a part of structures of urapidil and naftopidil (Figure 1) [39]. Urapidil, a well-known antihypertensive drug, acts as an α_1_-AR antagonist and 5-HT_1A_ agonist [43], while naftopidil is an α_1_-AR blocker with 3-fold higher affinity for α_1D_- than for α_1A_-AR subtype, and is commonly used in the treatment of patients with LUTS/BPH [44].

**Figure 2:**
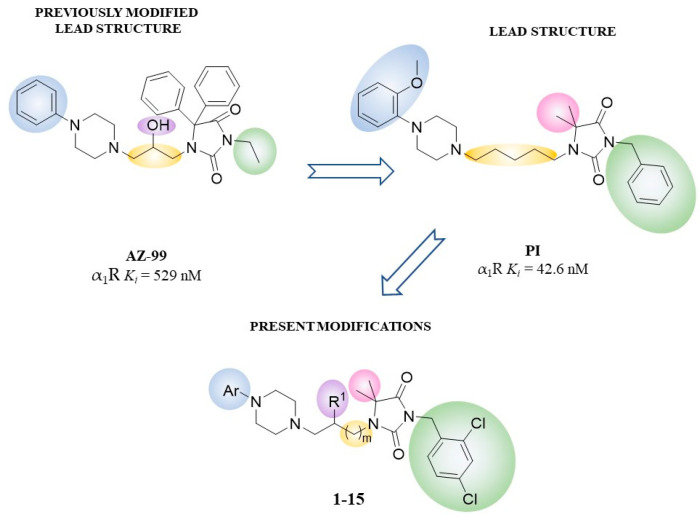
Structures of previously reported lead structure (**AZ-99**), the lead structure for the present study (**PI**), as well as the area of herein investigated structural modifications.

Our previous studies described syntheses and pharmacological properties of phenylpiperazine derivatives of phenytoin that displayed in vitro high affinity for α_1_-ARs and antagonistic properties in functional bioassays (Figure 2) [45,46,47]. Several compounds also showed antiarrhythmic effects via an α_1_-ARs dependent antagonistic action [41]. In addition, the derivative with o-methoxyphenylpiperazine moiety displayed hypotensive activity in an animal model [41]. The already described modifications included different types of substituents on piperazine, modification on linker (length and/or branching with hydroxy group) and modification on nitrogen atom N3 of hydantoin—ester or aliphatic chain (Figure 2). Nevertheless, we succeeded with the synthesis of compounds with better in vitro activity than reference **AZ-99**. Those compounds are characterized by high hydrophobic properties and moderate solubility. The continuation of this work led to the synthesis of compound **PI**, which was the first derivative without two aromatic rings in position 5 of hydantoin (two methyl groups instead) and with a benzyl group in position N3 (Figure 2) [48]. Based on structure-activity relationship (SAR) analysis from previous results and choosing compound **PI** as the lead structure, we designed the novel series of compounds (**1**–**15**, Table 1). In order to keep the balance between appropriate hydrophobicity and still meet the criteria of Barbaro’s pharmacophore model for α_1_-ARs activity [49], two chlorine atoms were introduced to the benzyl group (Figure 2). Moreover, the lead optimization concerned substituents on piperazine and linker type—length/(un)branching with the hydroxy group. Hence, the main goal of the present study was to investigate the impact of performed modifications on in vitro affinity for α_1_-ARs, as well as intrinsic activity at the α_1A_ and α_1B_ subtypes. Additionally, selected derivatives were investigated in vivo to determine an influence on arterial blood pressure in rats. The structural studies were enriched by X-ray crystallography and molecular docking to α_1_-ARs subtypes. 

## 2. Results

### 2.1. Chemistry

**Scheme 1:**
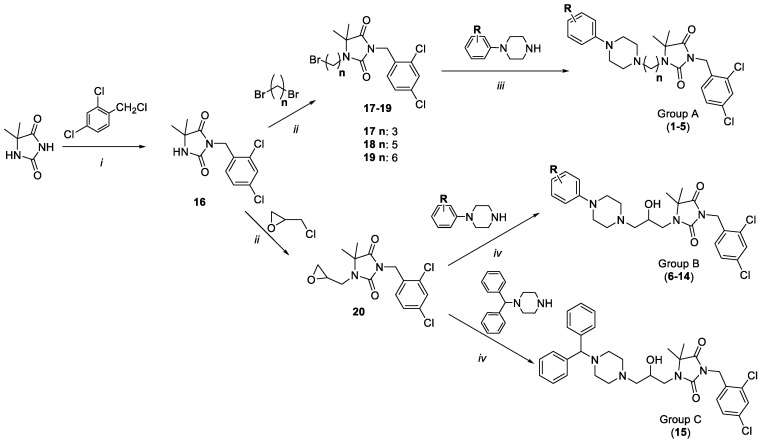
Syntheses of compounds **1**–**15**; (i) K_2_CO_3_, TEBA, acetone, reflux, 4 h; (ii) K_2_CO_3_, TEBA, acetone, rt, 120–168 h; (iii) K_2_CO_3_, acetone, reflux, 4–11 h; (iv) µw, 300–450 W, 3–7 min.

Syntheses of compounds **1**–**15** (Table 1) were performed according to Scheme 1.

Syntheses of compounds **7**–**9**, **15**, **16** and **18**–**20** were published before [48,50]. Syntheses of obtained compounds consisted of (i) N3-alkylation, (ii) N1-alkylation, and (iii or iv) arylpiperazine N-alkylation with suitable alkylation agents (**17**–**20**). Firstly, 5,5-dimethylhydantoin was reacted with 2,4-dichlorobenzyl chloride to obtain intermediate **16** (i, Scheme 1). In the next reaction (ii), N1-alkylation was conducted using dibromoalkyl (**17**–**19**, Group A) or 2-(chloromethyl)oxirane (**20**, Group B and C). The final N-alkylation was carried out using 1-phenylpiperazine derivatives to obtain products from Group A (**1**–**5**, Scheme 1) and Group B (**6**–**14**, Scheme 1). However, conditions for those two groups were different. In Group A, the reaction was carried out using acetone and potassium carbonate, while for Group B, the reaction was conducted using microwave (µw) irradiation. In the case of compound **15** (Group C), intermediate **20** was reacted with 1-benzhydrylpiperazine under microwave conditions.

### 2.2. X-ray Crystallographic Studies

**Figure 3:**
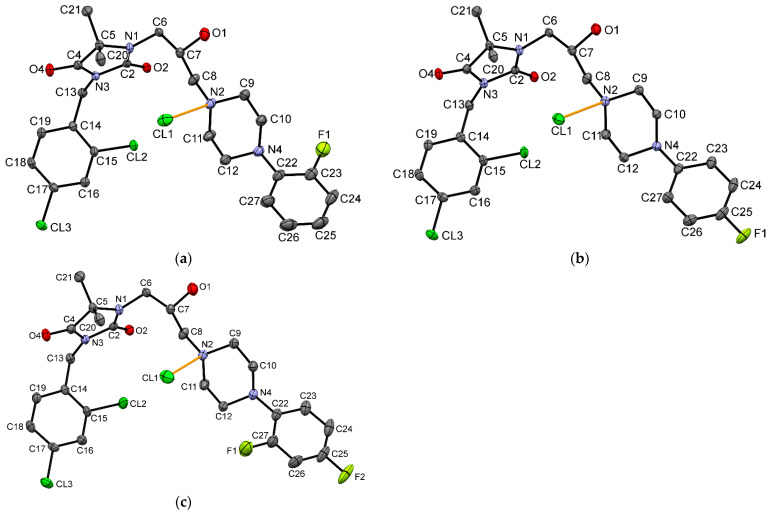
The molecular geometry of (**a**) **7**, (**b**) **8** and (**c**) **9**, with the atom numbering schemes. Hydrogen atoms have been omitted for clarity. Displacement ellipsoids are drawn at the 50% probability level. Orange lines show hydrogen bonds between the chloride anion and the protonated N2 atom.

The molecular geometry in the crystals of **7**, **8**, and **9** with the atom numbering schemes is shown in Figure 3.

**Figure 4:**
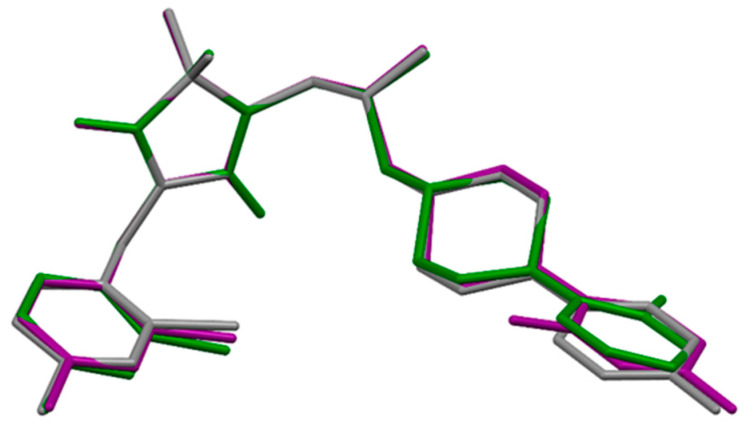
The overlap of the hydantoin rings of **7** (green), **8** (grey) and **9** (purple).

The overall shape of these three molecules is very similar (Figure 4). The linker, having a bent conformation, consists of three methylene units with a hydroxy group located at the second carbon atom. The oxygen atom of the hydroxy group is engaged in the intramolecular C9-H9···O1 hydrogen bond. Furthermore, the geometry of the linker is stabilized by intermolecular interactions, in which O-H···Cl and C-H···O hydrogen bonds are involved. Similar geometry was observed in another hydantoin derivative with the same linker [49].

Small differences are visible in the mutual orientation of hydantoin and aromatic rings, as well as hydantoin and piperazine rings. The piperazine ring adopts chair conformation with an equatorial position of substituents at N2 and N4 atoms. The investigated compounds have different substituents at the aromatic ring, which is connected to the piperazine ring, namely 2-F for **7**, 4-F for **8** and 2,4-di-F for **9**. The angle between the planes of the aromatic ring at the N4 atom and the carbon atoms of the piperazine ring is 49.86, 41.99 and 53.82° for **7**, **8** and **9**, respectively. The values of this angle for **7**, **8** are higher than in other crystal structures containing 4-(2-fluorobenzene)piperazine (the range of this angle 35.75–46.28°) or 4-(4-fluorobenzene)piperazine moiety (the range of this angle 11.52–35.68°) deposited in Cambridge Structure Database (CSD) [51]. There is no deposited crystal structure containing 4-(2,4-difluorobenzene)piperazine moiety in the CSD, which is present in **9**. The introduction of the second fluorine atom into the aromatic ring increases the value of this interplanar angle. The biggest difference in the geometry of analyzed molecules is observed in the location of 2-fluoro substituent of **7** in comparison to 2-fluoro substituent of **9**; namely, these substituents are on opposite sides (Figure 4).

In the presented crystal structures, the chlorine anion is involved in a charge-assisted hydrogen bond with a protonated N2 atom (Figure 3). The intermolecular interactions in the crystals are dominated by N-H···Cl, O-H···Cl, C-H···Cl and C-H···O hydrogen bonds. Additionally, C-H···F contacts are observed for structures containing 4-F substituent.

### 2.3. Affinity for α_1_-Adrenoceptors

In the first step, all synthesized compounds (**1**–**15**) were tested in vitro for their affinity for α_1_-ARs on rat cerebral cortex by the radioligand binding assay using [^3^H]-prazosin as a specific radioligand. The affinity described by *K_i_* (nM) values are given in Table 2. A majority of compounds (**1**, **3**, **5**–**9**, **11**, **13**) presented high affinity for α_1_-ARs (*K_i_* ≤ 100 nM). These compounds were identified to be more potent than urapidil, but none was as potent as the reference antagonist (prazosin). Among the novel compounds, four derivatives (**2**, **4**, **12**, **14**) showed moderate affinity to α_1_-ARs (100 < *K_i_* ≤ 535 nM). The newly synthesized derivatives, excluding compounds **2**, **10**, and **15**, presented higher affinity for the α_1_-ARs, when compared to compound **AZ-99**.

### 2.4. Intrinsic Activity

In the next step, selected compounds (**1**–**3**, **5**–**9**, **11**–**14**) were tested in vitro for their antagonistic activity at the α_1_-AR subtypes using the cells expressing mitochondrially-targeted aequorin and one of the human adrenergic receptors α_1A_ or α_1B_. The intrinsic activity expressed as IC_50_ values is presented in Table 3. The subtype selectivity for α_1A_ and α_1B_ was calculated as IC_50(α1B-AR)_/IC_50(α1A-AR)_ and IC_50(α1A-AR)_/IC_50(α1B-AR)_ ratio, respectively.

**Figure 5:**
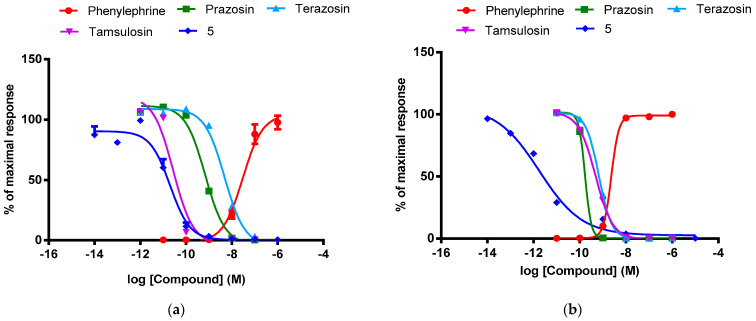
Concentration-response curves of phenylephrine, prazosin, terazosin, tamsulosin and compound **5** at the adrenergic (**a**) α_1A_ and (**b**) α_1B_ receptors. The values (%) are expressed as a percentage of the action of the reference agonist phenylephrine at the dose of EC_80_ (100%).

All tested compounds demonstrated antagonistic activity at the α_1_-AR subtypes. The strong (IC_50_ < 100 nM) or moderate (100 nM < IC_50_ < 200 nM) α_1A_-AR antagonistic intrinsic activity was observed for compounds **1** and **5**, or **9**, **11**, and **13**, respectively. Compound **5** was more potent at the α_1A_-AR than terazosin and prazosin and generally similar when compared to tamsulosin (Figure 5a). The strong α_1B_-AR antagonistic intrinsic activity (IC_50_ < 11 nM) was identified for compounds **1**, **3**, and **5**. Interestingly, compound **5** was more potent at the α_1B_-AR than all reference drugs: prazosin, terazosin and tamsulosin (Figure 5b). This compound was found to be the most potent α_1_-AR antagonist in the present group of derivatives of 5,5-dimethylhydantoin. In terms of subtype selectivity, compounds **5** and **3** displayed moderate (10-fold) and much higher (~90-fold) selectivity for α_1B_-AR over α_1A_-AR, respectively. Compounds **11** and **13** demonstrated slightly higher (~2-fold) selectivity for α_1A-AR_ over α_1B-AR_. The reference drugs, prazosin and terazosin, did not show subtype selectivity, while tamsulosin demonstrated moderate selectivity for α_1A_-AR (over α_1B_-AR), according to the literature data [52,53].

### 2.5. Molecular Modelling

**Figure 6:**
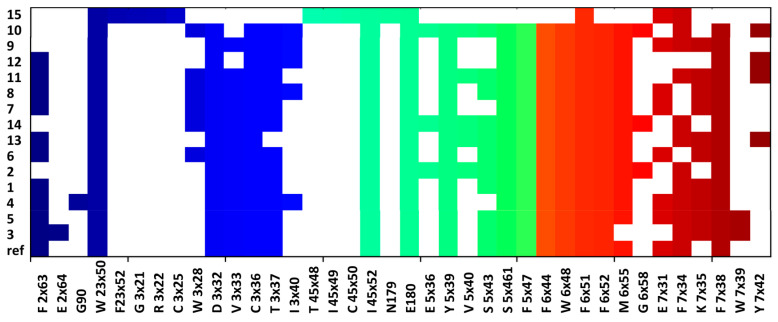
Ligand-protein interactions present in the compound complexes with α_1A_ receptors.

**Figure 7:**
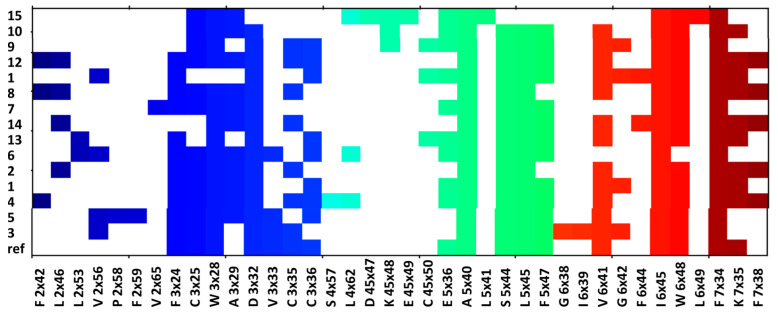
Ligand-protein interactions present in the compound complexes with α_1B_ receptors.

The compounds were docked to the inactive-state models of adrenergic receptors α_1A_ and α_1B_, constructed on the basis of the GPCRdb data. The ligand-protein contacts occurring in the obtained complexes are present in the form of the interaction matrices with α_1A_ (Figure 6) and α_1B_ receptors (Figure 7).

The contact patterns presented in both Figure 6 and Figure 7 show that the compounds consistently interact with the aspartic acid from the third transmembrane helix (TM3), D 3 × 32, for both α_1A_ and α_1B_ receptor subtype; the respective interaction is not present only for **15** (it is missing for both α_1A_ and α_1B_), which was characterized by the lowest affinity to α_1_ receptor (*K_i_* = 2443 nM).

In addition, all the compounds make contact with two residues from the second extracellular loop (ECL2): I 45 × 52, E180, and also with F × 51 when α_1A_ is taken into account and with A 5 × 40, I 6 × 45, and F 7 × 34, when α_1B_ is considered. 

In general, the interaction matrices show that the compounds adopted similar poses in the receptor binding sites: they consistently interact with the same set of residues from TM3, TM5, TM6, and TM7.

**Figure 8:**
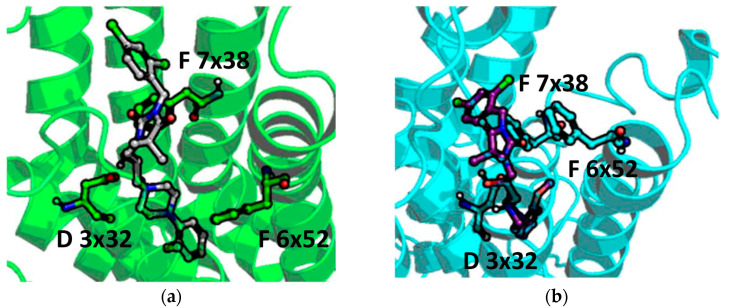
Docking results of **13** to (**a**) α_1A_ and (**b**) α_1B_.

The comparison of **13** (significantly better binder of α_1A_) position in α_1A_ a and α_1B_ (Figure 8) revealed that although the position of piperazine is similar for both receptor subtypes, the hydrogen bond with D 3 × 32 is formed only with α_1A_. Although compound **13** makes contact with D 3 × 32, the protonated nitrogen from the piperazine moiety is too far from the oxygen from the aspartic acid to make contact via a strong hydrogen bond. In addition, the methoxy moiety points towards TM5 for α_1A_, whereas in **13**-α_1B_ complex is oriented with the closest proximity to TM6.

**Figure 9:**
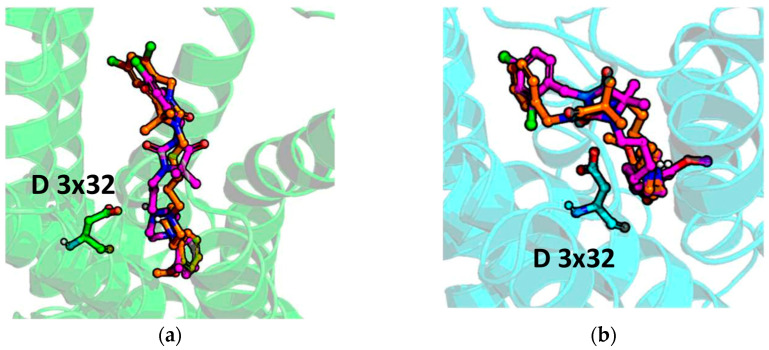
Docking results of **PI** (orange) and **3** (magenta) to (**a**) α_1A_ and (**b**) α_1B_.

Examining the ligand-protein interactions occurring for **PI** and **3** (Figure 9), it appears that the compound orientation in the binding site of both α_1A_ and α_1B_ is similar, and the piperazine moieties (forming the strongest interaction with the receptor) are almost aligned in both cases, which can explain the similar activity profile of **PI** and compound **3**. The introduction of di-Cl into the benzyl ring did not influence the overall compound pose but resulted in the formation of the additional interactions with W 7 × 39 in the case of α_1A_, and V 2 × 56 for α_1B_. However, as the di-Cl substitution faces towards extracellular part of the receptor, it probably does not influence compound affinity to such an extent, and the contact pattern of non-substituted **PI** is strong enough to provide the strong compound binding.

**Figure 10:**
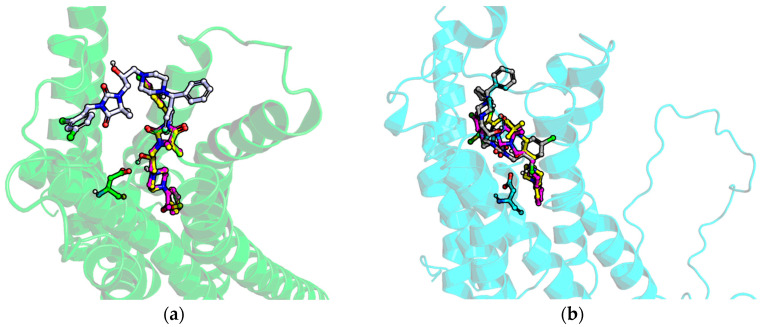
Docking results of **15** (grey), **13** (yellow), and **3** (magenta) to (**a**) α_1A_ and (**b**) α_1B_.

The low affinity of **15** is also reflected in docking studies, in which it adopted a flipped position in comparison to all remaining compounds for both α_1A_ and α_1B_ (Figure 10). As a result, the piperazine in **15** is located too far from the D 3 × 32 residue, and the hydrogen bond between the protonated nitrogen of piperazine and aspartic acid from TM3 cannot be formed. Instead, the piperazine interacts with E180 from ECL2 for α_1A_, and K 45 × 48, E 45 × 49, C 45 × 50 for α_1B_; however, the overall position of **15** in the receptor binding site (for both α_1A_, and α_1B_) is very shallow (especially for α_1A_) and does not provide sufficient contact network to strongly fit the compound in the binding pocket.

### 2.6. The Influence on Blood Pressure in Rats

**Figure 11:**
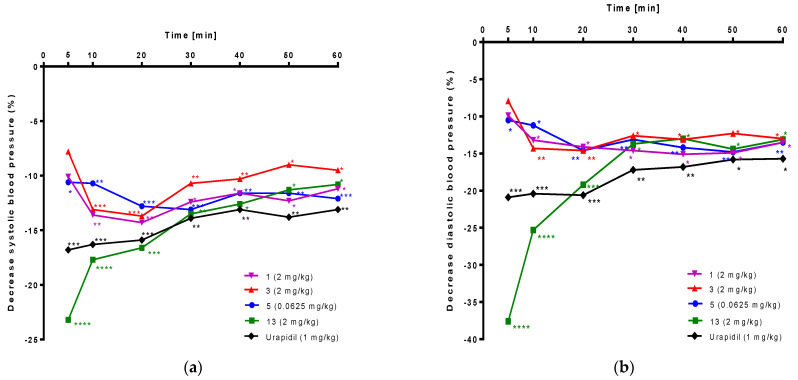
Hypotensive activity of **1**, **3**, **5**, **13**, and urapidil tested after i.v. administration to anesthetized normotensive rats. Results are expressed as a percentage of decrease (**a**) systolic and (**b**) diastolic blood pressure. Statistical analysis: two-way ANOVA and Sidak’s multiple comparisons test. Statistically significant: * *p* < 0.05, ** *p* < 0.02, *** *p* < 0.01, **** *p* < 0.001, vs. control group (vehicle treatment); n = 6 rats per each group.

In the next step, the hypotensive activity of novel compounds **1**–**3**, **5**–**9**, and **11**–**14** was determined after i.v. administration to normotensive anesthetized rats. All results are shown in Supplementary Table S1. Four compounds (**1**, **3**, **5**, **13**) proved statistically significant reduction of systolic and diastolic blood pressure. For these compounds, the hypotensive activity expressed as a percentage of decreased systolic and diastolic blood pressure is displayed in Figure 11a and Figure 11b, respectively.

Compound **5** administered at the dose 0.0625 mg/kg, significantly reduced systolic blood pressure by 13.1–10.6% and diastolic blood pressure by 14.8–10.5% from the fifth minute of the observation period. Compound **13** at the dose 2.0 mg/kg, significantly decreased systolic blood pressure by 23.2–10.8% and diastolic blood pressure by 37.6–13.0% from the fifth minute after administration. Compound **3** at the dose 2.0 mg/kg, significantly reduced systolic blood pressure by 13.7–9.0% and diastolic blood pressure by 14.6–12.3% from the tenth minute after administration. Compound **1** at the dose 2.0 mg/kg, significantly reduced systolic blood pressure by 14.3–11.2% and diastolic blood pressure by 14.9–13.2% from the tenth minute of the observation period. Urapidil (used as a reference compound) at the lowest hypotensive dose (1.0 mg/kg) significantly decreased systolic blood pressure by 16.8–13.1% and diastolic blood pressure by 20.9–15.7% from the fifth minute after administration. 

## 3. Discussion

The new group of 5,5-dimethylhydantoin derivatives (**1**–**15**) is characterized by modifications in two fragments: arylpiperazine and alkyl linker (Figure 2). The first one involves un-, mono- and disubstituted phenyl ring (Group A and B, Table 1) or diphenylmethyl moiety (Group C, Table 1). The other embraced either three different lengths of chain (n = 3, 5 or 6, Group A) or branching with hydroxyl group (Group B and C). The current design was based on a previously found active compound, **PI** (Figure 2), and accordingly, final products possess 5,5-dimethyl- fragment. Furthermore, the benzyl moiety connected to N3 position of hydantoin was slightly modified by the addition of two chlorine atoms to achieve proper hydrophobic properties. Taking into consideration previously obtained active compounds, described modifications of moieties attached to N1 position enabled to carry out structure—activity relationship (SAR) analysis of obtained compounds with potential influence on α_1_-ARs. 

Affinity values for α_1_-ARs were determined for all compounds (**1**–**15**) using the radioligand binding assay. Nine compounds (**1**, **3**, **5**–**9**, **11**, and **13**) displayed *K*_i_ lower than urapidil (Table 2). Among the four, the most potent compounds (**1**, **3**, **5**, **13**) proved to have high affinity for α_1_-ARs (*K_i_* = 31.0, 43.0, 12.7, 29.0 nM, respectively), three ones (**1**, **5**, **13**) contain an o-methoxyphenylpiperazine fragment in their structure. Moreover, compound **11,** possessing a cyano group at the *o*-position of the phenyl ring, also showed a high affinity for these receptors (*K*_i_ = 26.0 nM). Nevertheless, the most potent compound (**5**) possesses an o-methoxyphenylpiperazine fragment and hexyl linker at the N1-position of hydantoin. Moreover, current studies results proved that presence of chlorine in phenyl ring is not advantageous (**1** vs. **2**, **5** vs. **4**, and **6** vs. **10**, Table 2), while compounds containing fluorine atom(s) at position *ortho* and *para* (**9**) or *para* (**8**) at the phenyl ring of phenylpiperazine moiety displayed higher affinity for α_1_-ARs (*K*_i_ = 42.0, 44.0 nM, respectively) than compound **7** possessing an *o*-fluorophenylpiperazine moiety (*K*_i_ = 100 nM). The molecular geometry of these compounds in the crystals is very similar (Figure 4). Our research also indicated that the substituents at position *meta*, *ortho* and *meta* or *meta* and *para* of the phenylpiperazine phenyl ring seem to be unfavorable. The obtained results are in agreement with the previously drawn conclusion that the methoxy group at *o*-position at the phenyl ring of phenylpiperazine moiety is favorable for the affinity to α_1_-ARs [39,41,42,45,47,54,55,56,57]. In the case of the linker, a long carbon chain consisting of 6 carbon atoms seems to have a beneficial influence on affinity to α_1_-ARs of an o-methoxyphenylpiperazine derivative. 

The intrinsic activity assay enabled the determination of agonist/antagonist profile as well as selectivity towards α_1A_- and α_1B_-ARs. All selected compounds (**1**–**3**, **5**–**9**, **11**–**14**) showed antagonistic activity to both tested α_1_-AR subtypes (Table 3). Two compounds (**1** and **5**), both belonging to group A and possessing methoxy group at o-position at the phenyl ring, displayed strong (IC_50_ < 100 nM) α_1A_-AR antagonistic intrinsic activity, while representatives from group B (**9**, **11**, **13**) proved moderate one (100 nM < IC_50_ < 200 nM). In the case of α_1B_-AR, all o-methoxyphenylpiperazine derivatives from group A (**1** and **5**) showed IC_50_ < 100 nM. Nevertheless, selectivity ratio calculation revealed that 2-methoxy (**13**) and 2-cyano (**11**) group with 2-hydroksypropyl linker at the N1-position of hydantoin could be responsible for better subtype selectivity for α_1A_ (over α_1B_), while long carbon chains, first of all hexyl (**5**), secondly pentyl (**3**), linkers seem to be beneficial for the subtype selectivity for α_1B_ (over α_1A_). Furthermore, the antagonistic intrinsic activity of **5** towards α_1A_ was similar to the most potent tested reference, tamsulozin (Figure 5a) and higher than all reference compounds in the case of α_1B_-AR, including tamsulozin (Figure 5b). 

In vivo assay on normotensive rats was carried out in order to assess the hypotensive activity of selected compounds (**1**–**3**, **5**–**9**, **11**–**14**). All tested compounds possessing o-methoxyphenylpiperazine fragments (**1**, **5**, **13**) proved significant reduction of systolic and diastolic blood pressure as well as one with unsubstituted phenylpiperazine (**3**). Furthermore, these effects were observed for compound **5** in a 16-fold lower dose than for urapidil (0.0625 vs. 1.0 mg/kg, respectively). Results obtained via in vivo study correlated with in vitro functional assays. The hypotensive activity compounds showed strong antagonistic effects at α_1A_-AR (**13**) and/or α_1B_-AR (**1**, **3**, **5**), but the short-term, strong hypotensive effect of compound **13** may result from a different mechanism of antihypertensive action than the α_1_-blocker or different pharmacokinetics, which would be worth investigating at a later stage of research. Moreover, compound **5** displayed a stronger effect at α_1_-ARs than classic α_1_-AR blockers like prazosin and terazosin, well known as antihypertensive drugs [58,59]. The presented results suggested that these compounds exhibited hypotensive activity through α_1_-AR blockade. Analyzing the structure of the most active compound (**5**), it could be stated that methoxy-substituent at the ortho position of the phenyl ring and hexyl linker at the N1-position of hydantoin are very favorable in case of hypotensive effects by blocking α_1B_-AR. The identified α_1A_-AR selective antagonists also can help the design of efficacious drugs for arrhythmias. Discovery of new α_1_-AR antagonist (**5**), moderately selective for α_1B_ over α_1A_ subtype (10-fold), is promising for the development of novel hypotensive drugs. Moreover, among tested phenylpiperazine derivatives, compound **11** (o-CN-phenylpiperazine derivative) exhibited no significant changes in the hemodynamic parameters (SBP and DBP) and demonstrated in vitro strong antagonistic activity at α_1A_-AR. Moreover, this compound showed a slight selectivity for α_1A_ over α_1B_ subtype. This can suggest that 2-cyano- substituent at the phenyl ring of the phenylpiperazine moiety exerts a beneficial influence on α_1A_ subtype selectivity (over α_1B_). Thus, these data can aid the development of new α_1A_-AR selective blockers used in the treatment of patients with BPH/LUTS.

## 4. Materials and Methods

### 4.1. Chemistry

Reagents were purchased from Alfa Aesar (Karlsruhe, Germany) or Sigma Aldrich (Darmstadt, Germany). Reaction progress was verified using thin layer chromatography (TLC), which was carried out on 0.2 mm Merck silica gel 60 F254 plates. Spots were visualized by UV light. Melting points (m.p.) were determined using the MEL-TEMP II apparatus (LD Inc., Long Beach, CA, USA) and were uncorrected. The ^1^H-NMR spectra were obtained on a Mercury-VX 300 Mz spectrometer (Varian, Palo Alto, CA, USA) in DMSO-d6. Chemical shifts in ^1^H-NMR spectra were reported in parts per million (ppm) on the δ scale using the solvent signal as an internal standard. Data are reported as follows: chemical shift, multiplicity (s, singlet; d, doublet; dd—doublet of doublets; t, triplet; m, multiplet), coupling constant J in Hertz (Hz), number of protons, proton’s position (Im—imidazolone, Benz- benzyl, Ph—phenyl, Pp—piperazine). Mass spectra were recorded on a UPLC-MS/MS system consisting of a Waters ACQUITY^®^UPLC^®^ (Waters Corporation, Milford, MA, USA) coupled to a Waters TQD mass spectrometer (electrospray ionization mode ESI-tandem quadrupole). Chromatographic separations were carried out using the Acquity UPLC BEH (bridged ethyl hybrid) C18 column; 2.1 × 100 mm, and 1.7 µm particle size, equipped with Acquity UPLC BEH C18 VanGuard precolumn (Waters Corporation, Milford, MA, USA); 2.1 × 5 mm, and 1.7 µm particle size. The column was maintained at 40 °C and eluted under gradient conditions from 95% to 0% of eluent A over 10 min, at a flow rate of 0.3 mL·min−1. Eluent A: water/formic acid (0.1%, *v/v*); eluent B: acetonitrile/formic acid (0.1%, *v/v*). Chromatograms were made using Waters eλ PDA detector. Spectra were analyzed in the 200–700 nm range with 1.2 nm resolution and a sampling rate 20 points/s. MS detection settings of Waters TQD mass spectrometer were as follows: source temperature 150 °C, desolvation temperature 350 °C desolvation gas flow rate 600 L·h^−1^, cone gas flow 100 L·h^−1^, capillary potential 3.00 kV, cone potential 40 V. Nitrogen was used for both nebulizing and drying gas. The data were obtained in a scan mode ranging from 50 to 1000 *m/z* in time 0.5 s intervals. The data acquisition software was MassLynx V 4.1 (Waters Corporation, Milford, MA, USA). The UPLC/MS purity of all the final compounds was confirmed to be 95% or higher. Retention times (t_R_) are given in minutes. The UPLC/MS purity of all final compounds was determined (%). Syntheses under microwave irradiation were performed in a household microwave oven Samsung MW71B. Synthesis of compounds **7**–**9**, **15**, **16**, **18**–**20** was described earlier [48,50], and the procedure for intermediate **17** can be found in Supplementary Materials.

#### 4.1.1. General Procedure to Obtain Final Products from Group A (**1**–**5**)

Piperazine derivatives (2–4 mmol), potassium carbonate (6.5–13.0 mmol, 0.9–1.80 g), acetone (7.5–15.0 mL), and 2,4-dichlorobenzyl-1-bromoalkyl-5,5-dimethylimidazolidine-2,4-dione derivatives (**17**–**19**) (4.0–8.0 mmol) dissolved in acetone (10.0–15.0 mL) were heated at reflux for 3.75–11 h. Then, filtration was carried out. The filtrate was evaporated. To the residue, dichloromethane was added, and it was washed 2–3 times with 1% HCl. Organic fractions were dried, then filtrated and evaporated. Solid products were obtained using method A-B. Method A—Saturation using gaseous hydrochloric acid; Method B—When method A was unsuccessful, the obtained compound was dissolved in anhydrous ethanol, and diethyl ether was added. It was mixed for 30 min and then put into the refrigerator for 2–7 days. Then, filtration was performed. Method C: If the solid product from method A or B was impure, it was heated in acetone for 5 min. The suspension was put into the fridge for 15 min and then into the refrigerator for an additional 15 min. Finally, Buchner filtration was conducted to obtain a pure product.

3-(2,4-dichlorobenzyl)-1-(3-(4-(2-methoxyphenyl)piperazin-1-yl)propyl)-5,5-dimethylimidazolidine-2,4-dione hydrochloride (**1**).

1-(2-methoxyphenyl)piperazine (4 mmol, 0.77 g) and the 3-bromopropyl derivative (**17**) (8.0 mmol, 3.2 g) were used. Product was obtained using method A. White solid. Yield 60.0%; mp 201–203 °C. C_26_H_33_Cl_3_N_4_O_2_ MW 555.93. LC/MS±: purity 98.64% t_R_ = 5.60, (ESI) *m/z* [M + H] 519.12. ^1^H NMR (DMSO-d6, ppm): δ 10.67 (s, 1H, NH^+^), 7.66 (d, *J* = 2.1 Hz, 1H, Benz-3-H), 7.43 (dd, *J* = 8.4, 2.2 Hz, 1H, Benz-5-H), 7.21 (d, *J* = 8.4 Hz, 1H, Benz-6-H), 7.07–6.86 (m, 4H, PpPh-3,4,5,6-H), 4.63 (s, 2H, N3-CH_2_), 3.79 (s, 3H, OCH_3_), 3.60–3.34 (m, 6H, Pp-3,5-H, N1-CH_2_), 3.25–2.97 (m, 6H, Pp-2,6-H, Pp-CH_2_), 2.13–1.95 (m, 2H, Pp-CH_2_-CH_2_), 1.42 (s, 6H, 2 × Im-CH_3_).

3-(2,4-dichlorobenzyl)-1-(3-(4-(2,3-dichlorophenyl)piperazin-1-yl)propyl)-5,5-dimethylimidazolidine-2,4-dione hydrochloride (**2**).

1-(2,3-dichlorophenyl)piperazine (4 mmol, 0.92 g) and the 3-bromopropyl derivative (**17**) (8.0 mmol, 3.2 g) were used. The product was obtained using method B. White solid. Yield 62.0%; mp 185–187 °C. C_25_H_29_Cl_5_N_4_O_2_ MW 594.78. LC/MS±: purity 97.84% t_R_ = 6.48, (ESI) *m/z* [M + H] 559.00. ^1^H NMR (DMSO-d6, ppm): δ 10.73 (s, 1H, NH^+^), 7.64 (d, *J* = 2.1 Hz, 1H, Benz-3-H), 7.44–7.29 (m, 3H, Benz-5,6-H, PpPh-4-H), 7.23–7.17 (m, 2H, PpPh-5,6-H), 4.61 (s, 2H, N3-CH_2_), 3.56 (d, *J* = 5.9 Hz, 2H, Pp-3,5-H_b_), 3.47–3.31 (m, 4H, N1-CH_2_, Pp-3,5-H_a_), 3.25–3.05 (m, 6H, Pp-CH_2_, Pp-2,6-H), 2.12–1.92 (m, 2H, Pp-CH_2_-CH_2_), 1.38 (s, 6H, 2 × Im-CH_3_).

3-(2,4-dichlorobenzyl)-5,5-dimethyl-1-(5-(4-phenylpiperazin-1-yl)pentyl)imidazolidine-2,4-dione hydrochloride (**3**).

1-phenylpiperazine (4 mmol, 0.65 g) and the 3-bromopentyl derivative (**18**) (5 mmol, 2.18 g) were used. The product was obtained using method A. White solid. Yield 37.5%; mp 218–220 °C. C_27_H_35_Cl_3_N_4_O_2_ MW 553.95. LC/MS±: purity 100.00% t_R_ = 5.90, (ESI) *m/z* [M + H] 517.13. ^1^H NMR (DMSO-d6, ppm): δ 10.49 (s, 1H, NH^+^), 7.65 (d, *J* = 2.1 Hz, 1H, Benz-3-H), 7.43 (dd, *J* = 8.4, 2.2 Hz, 1H, Benz-5-H), 7.31–7.22 (m, 2H, PpPh-3,5-H), 7.18 (d, *J* = 8.4 Hz, 1H, Benz-6-H), 7.00 (d, *J* = 7.9 Hz, 2H, PpPh-2,6-H), 6.86 (t, *J* = 7.3 Hz, 1H, PpPh-4-H), 4.62 (s, 2H, N3-CH_2_), 3.88–3.44 (m, 4H, Pp-3,5-H), 3.28 (t, *J* = 7.4 Hz, 2H, N1-CH_2_), 3.19–3.00 (m, 6H, Pp-CH_2_, Pp-2,6-H), 1.83–1.68 (m, 2H, N1-CH_2_CH_2_), 1.68–1.54 (m, 2H, Pp-CH_2_CH_2_), 1.38 (s, 6H, 2 × Im-CH_3_), 1.36–1.29 (m, 2H, PpCH_2_CH_2_CH_2_).

3-(2,4-dichlorobenzyl)-1-(6-(4-(3,4-dichlorophenyl)piperazin-1-yl)hexyl)-5,5-dimethylimidazolidine-2,4-dione hydrochloride (**4**).

1-(3,4-dichlorophenyl)piperazine (2 mmol, 0.46 g) and the 3-bromohexyl derivative (**19**) (4.0 mmol, 1.80 g) were used. Product was obtained using methods B and C. White solid. Yield 2.0%; mp 154–156 °C. C_28_H_35_Cl_5_N_4_O_2_ MW 636.86. LC/MS±: purity 99.10% t_R_ = 6.84, (ESI) *m/z* [M + H] 601.46. ^1^H NMR (DMSO-d6, ppm): δ 11.09 (s, 1H, NH^+^), 7.64 (d, *J* = 2.1 Hz, 1H, Benz-3-H), 7.50–7.37 (m, 2H, Benz-5-H, Ph-5-H), 7.24 (d, *J* = 2.8 Hz, 1H, Ph-2-H), 7.17 (d, *J* = 8.4 Hz, 1H, Benz-6-H), 7.00 (dd, *J* = 9.0, 2.8 Hz, 1H, Ph-6-H), 4.61 (s, 2H, N3-CH_2_), 3.95–3.80 (m, 2H, Pp-3,5-H_b_), 3.58–3.43 (m, 2H, Pp-3,5-H_a_), 3.35–3.16 (m, 4H, N1-CH_2,_ Pp-CH_2_), 3.14–2.97 (m, 4H, Pp-2,6-H), 1.84–1.66 (m, 2H, Pp-CH_2_CH_2_), 1.66–1.50 (m, 2H, N1-CH_2_CH_2_), 1.44–1.26 (m, 10H, N1-CH_2_CH_2_CH_2,_ Pp-CH_2_CH_2_CH_2,_ 2 × Im-CH_3_).

3-(2,4-dichlorobenzyl)-1-(6-(4-(2-methoxyphenyl)piperazin-1-yl)hexyl)-5,5-dimethylimidazolidine-2,4-dione hydrochloride (**5**).

1-(2-metoxyphenyl)piperazine (2 mmol, 0.38 g) and the 3-bromohexyl derivative (**19**) (4.0 mmol, 1.80 g) were used. Product was obtained using methods A and C. White solid. Yield 45.0%; mp 205–207 °C. C_29_H_39_Cl_3_N_4_O_3_ MW 598.01. LC/MS±: purity 95.67% t_R_ = 6.19, (ESI) *m/z* [M + H] 562.26. ^1^H NMR (DMSO-d6, ppm): δ 11.02 (s, 1H, NH^+^), 7.65 (d, *J* = 2.1 Hz, 1H, Benz-3-H), 7.43 (dd, *J* = 8.3, 2.2 Hz, 1H, Benz-5-H), 7.17 (d, *J* = 8.4 Hz, 1H, Benz-6-H), 7.06–6.87 (m, 4H, PpPh-3,4,5,6-H), 4.62 (s, 2H, N3-CH_2_), 3.80 (s, 3H, OCH_3_), 3.58–3.39 (m, 4H, Pp-3,5-H), 3.31–3.20 (m, 2H, N1-CH_2_), 3.20–3.00 (m, 6H, Pp-CH_2_, Pp-2,6-H), 1.82–1.66 (m, 2H, Pp-CH_2_CH_2_), 1.66–1.50 (m, 2H, N1-CH_2_CH_2_), 1.45–1.26 (m, 10H, N1-CH_2_CH_2_CH_2,_ Pp-CH_2_CH_2_CH_2_, 2 × Im-CH_3_).

#### 4.1.2. General Procedure to Obtain Final Products from Group B (**6**, **10**–**14**)

Piperazine derivatives (3 mmol) and the 3-(2,4-dichlorobenzyl)-5,5-dimethyl-1-(oxiran-2-ylmethyl)imidazolidine-2,4-dione (**20**) (3 mmol) were dissolved in acetone (15–20 mL). The mixture was evaporated and irradiated in a household microwave (300–450 W) for 3–7 min. Crystallization using ethanol anhydrous was conducted. Method A: Solid products were obtained through saturation using gaseous hydrochloric acid. Method B: When method A was unsuccessful, the obtained compound was dissolved in anhydrous ethanol, and diethyl ether was added. It was stirred for 30 min and then put into the refrigerator for 2–7 days. Then, filtration was carried out. In order to obtain pure product, crystallization from acetone was carried out.

3-(2,4-dichlorobenzyl)-1-(2-hydroxy-3-(4-phenylpiperazin-1-yl)propyl)-5,5-dimethylimidazolidine-2,4-dione hydrochloride (**6**).

1-phenylpiperazine (3 mmol, 0.49 g) and 3-(2,4-dichlorobenzyl)-5,5-dimethyl-1-(oxiran-2-ylmethyl)imidazolidine-2,4-dione (**20**) (3.0 mmol, 1.03 g) were used. The product was obtained using method B. White solid. Yield 89.0%; mp 196–198 °C. C_25_H_31_Cl_3_N_4_O_3_ MW 541.90. LC/MS±: purity 99.45% t_R_ = 6.60, (ESI) *m/z* [M + H] 506.44. ^1^H NMR (DMSO-d6, ppm): δ 10.74 (s, 1H, NH^+^), 7.64 (d, *J* = 2.1 Hz, 1H, Benz-3-H), 7.42 (dd, *J* = 8.4, 2.2 Hz, 1H, Benz-5-H), 7.31–7.21 (m, 3H, Benz-6-H, Ph-3,5-H), 7.00 (d, *J* = 7.9 Hz, 2H, Ph-2,6-H), 6.86 (t, *J* = 7.3 Hz, 1H, Ph-4-H), 4.63 (s, 2H, N3-CH_2_), 4.45–4.33 (m, *J* = 7.3 Hz, 1H, CH-OH), 3.93–3.01 (m, 12H, Pp-2,3,5,6-H, Pp-CH_2,_ N1-CH_2_), 1.44 (s, 3H, 2 × Im- CH_3b_), 1.41 (s, 3H, 2 × Im-CH_3a_).

3-(2,4-dichlorobenzyl)-1-(3-(4-(3,4-dichlorophenyl)piperazin-1-yl)-2-hydroxypropyl)-5,5-dimethylimidazolidine-2,4-dione hydrochloride (**10**).

1-(3,4-dichlorophenyl)piperazine (3 mmol, 0.69 g) and 3-(2,4-dichlorobenzyl)-5,5-dimethyl-1-(oxiran-2-ylmethyl)imidazolidine-2,4-dione (**20**) (3.0 mmol, 1.03 g) were used. The product was obtained using method A. White solid. Yield 4.0%; mp 223–225 °C. C_25_H_29_Cl_5_N_4_O_3_ MW 610.78. LC/MS±: purity 97.61% t_R_ = 6.29, (ESI) *m/z* [M + H] 575.33. ^1^H NMR (DMSO-d6, ppm): δ 10.56 (s, 1H, NH^+^), 7.65 (d, *J* = 2.0 Hz, 1H, Benz-3-H), 7.50–7.38 (m, 2H, Benz-5-H, PpPh-5-H), 7.31–7.21 (m, 2H, Benz-6-H, PpPh-2-H), 7.00 (dd, *J* = 9.0, 2.5 Hz, 1H, PpPh-6-H), 5.94 (s, 1H, OH), 4.63 (s, 2H, N3-CH_2_), 4.48–4.24 (m, 1H, CH-OH), 4.04–3.77 (m, 2H, N1-CH_2_), 3.75–3.35 (m, 4H, Pp-3,5-H), 3.30–2.97 (m, 6H, Pp-2,6-H, Pp-CH_2_), 1.43 (s, 3H, Im-CH_3b_), 1.41 (s, 3H, Im-CH_3a_).

3-(2,4-dichlorobenzyl)-1-(3-(4-(2-cyanophenyl)piperazin-1-yl)-2-hydroxypropyl)-5,5-dimethylimidazolidine-2,4-dione hydrochloride (**11**).

1-(2-cyanophenyl)piperazine (3 mmol, 0.56 g) and 3-(2,4-dichlorobenzyl)-5,5-dimethyl-1-(oxiran-2-ylmethyl)imidazolidine-2,4-dione (**20**) (3.0 mmol, 1.03 g) were used. The product was obtained using method A. Cream solid. Yield 25.0%; mp 147–149 °C. C_26_H_30_Cl_3_N_5_O_3_ MW 566.91. LC/MS±: purity 100.00% t_R_ = 5.46, (ESI) *m/z* [M + H] 531.45. 

^1^H NMR (DMSO-d6, ppm): δ 10.66 (s, 1H, NH^+^), 7.76 (dd, *J* = 7.7, 1.5 Hz, 1H, PpPh-3-H), 7.69–7.60 (m, 2H, PpPh-5-H, Benz-3-H), 7.43 (dd, *J* = 8.4, 2.2 Hz, 1H, Benz-5-H), 7.26 (m, 2H, Benz-6-H, PpPh-6-H), 7.18 (t, *J* = 7.6 Hz, 1H, PpPh-4-H), 5.94 (s, 1H, OH), 4.64 (s, 2H, N3-CH_2_), 4.38 (s, 1H, CH-OH), 3.81–3.50 (m, 4H, N1-CH_2,_ Pp-3,5-H_a_), 3.46–3.04 (m, 8H, Pp-3,5-H_b,_ Pp-2,6-H, Pp-CH_2_), 1.44 (s, 3H, Im-CH_3b_), 1.42 (s, 3H, Im-CH_3a_).

3-(2,4-dichlorobenzyl)-1-(3-(4-(3,4-dimethylphenyl)piperazin-1-yl)-2-hydroxypropyl)-5,5-dimethylimidazolidine-2,4-dione hydrochloride (**12**).

1-(3,4-dimethylphenyl)piperazine (3 mmol; 0.57 g) and 3-(2,4-dichlorobenzyl)-5,5-dimethyl-1-(oxiran-2-ylmethyl)imidazolidine-2,4-dione (**20**) (3.0 mmol; 1.03 g) were used. The product was obtained using method A. White solid. Yield 89.0%; mp 217–219 °C. C_27_H_35_Cl_3_N_4_O_3_ MW 569.95. LC/MS±: purity 97.44% t_R_ = 5.92; (ESI) *m/z* [M + H] 534.49. ^1^H NMR (DMSO-d6; ppm): δ 10.56 (s; 1H; NH^+^), 7.64 (d; *J* = 2.1 Hz; 1H; Benz-3-H); 7.42 (dd; *J* = 8.4; 2.2 Hz; 1H; Benz-5-H); 7.25 (d; *J* = 8.4 Hz; 1H; Benz-6-H); 7.00 (d; *J* = 8.3 Hz; 1H; PpPh-5-H); 6.80 (d; *J* = 2.3 Hz; 1H; PpPh-2-H); 6.70 (dd; *J* = 8.2; 2.5 Hz; 1H; PpPh-6-H); 6.07–5.85 (m; 1H; OH); 4.63 (s; 2H; N3-CH_2_); 4.45–4.31 (m; 1H; CH-OH); 3.85–2.99 (m; 12H; N1-CH_2;_ Pp-2,3,5,6-H; Pp-CH_2_); 2.19 (s; 3H; PpPh-3-CH_3_); 2.11 (s; 3H; PpPh-4-CH_3_); 1.44 (s; 3H; Im-CH_3b_); 1.42 (s; 3H; Im-CH_3a_).

3-(2,4-dichlorobenzyl)-1-(2-hydroxy-3-(4-(2-methoxyphenyl)piperazin-1-yl)propyl)-5,5-dimethylimidazolidine-2,4-dione hydrochloride (**13**).

1-(2-metoxyphenyl)piperazine (3 mmol, 0.58 g) and 3-(2,4-dichlorobenzyl)-5,5-dimethyl-1-(oxiran-2-ylmethyl)imidazolidine-2,4-dione (**20**) (3.0 mmol, 1.03 g) were used. Product was obtained using method A. White solid. Yield 36.0%; mp 144–146 °C. C_26_H_33_Cl_3_N_4_O_4_ MW 571.92. LC/MS±: purity 97.54% t_R_ = 5.48, (ESI) *m/z* [M + H] 536.46. ^1^H NMR (DMSO-d6, ppm): δ 10.62 (s, 1H, NH^+^), 7.65 (d, *J* = 2.1 Hz, 1H, Benz-3-H), 7.42 (dd, *J* = 8.4, 2.1 Hz, 1H, Benz-5-H), 7.26 (d, *J* = 8.4 Hz, 1H, Benz-6-H), 7.07–6.86 (m, 4H, PpPh-3,4,5,6-H), 5.86 (s, 1H, OH), 4.63 (s, 2H, N3-CH_2_), 4.46–4.30 (m, 1H, CH-OH), 3.79 (s, 3H, OCH_3_), 3.73–3.01 (m, 12H, N1-CH_2,_ Pp-2,3,5,6-H, Pp-CH_2_), 1.45 (s, 3H, Im-CH_3b_), 1.42 (s, 3H, Im-CH_3a_).

3-(2,4-dichlorobenzyl)-1-(2-hydroxy-3-(4-(3-methoxyphenyl)piperazin-1-yl)propyl)-5,5-dimethylimidazolidine-2,4-dione hydrochloride (**14**).

1-(3-metoxyphenyl)piperazine (3 mmol, 0.58 g) and 3-(2,4-dichlorobenzyl)-5,5-dimethyl-1-(oxiran-2-ylmethyl)imidazolidine-2,4-dione (**20**) (3.0 mmol, 1.03 g) were used. The product was obtained using method A. White solid. Yield 38.0%; mp 177–179 °C. C_26_H_33_Cl_3_N_4_O_4_ MW 571.92. LC/MS±: purity 100.00% t_R_ = 5.45, (ESI) *m/z* [M + H] 536.46. ^1^H NMR (DMSO-d6, ppm): δ 10.59 (s, 1H, NH^+^), 7.65 (d, *J* = 2.1 Hz, 1H, Benz-3-H), 7.42 (dd, *J* = 8.4, 2.2 Hz, 1H, Benz-5-H), 7.25 (d, *J* = 8.4 Hz, 1H, Benz-6-H), 7.15 (t, *J* = 8.2 Hz, 1H, PpPh-5-H), 6.57 (dd, *J* = 8.2, 2.0 Hz, 1H, PpPh-6-H), 6.52 (t, *J* = 2.2 Hz, 1H, PpPh-2-H), 6.44 (dd, *J* = 8.1, 2.0 Hz, 1H, PpPh-4-H), 6.02–5.89 (m, 1H, OH), 4.63 (s, 2H, N3-CH_2_), 4.45–4.31 (m, 1H, CH-OH), 3.91–3.00 (m, 15H, OCH_3,_ N1-CH_2,_ Pp-2,3,5,6-H, Pp-CH_2_), 1.44 (s, 3H, Im-CH_3b_), 1.42 (s, 3H, Im-CH_3a_).

### 4.2. Crystallography

Crystals suitable for an X-ray structure analysis were grown from n-butyl acetate for **7**, while for **8** and **9** from n-propyl acetate, by slow evaporation of the solvent at room temperature.

Data for single crystals of **7** and **9** were collected at 130K using the Oxford Diffraction SuperNova four-circle diffractometer equipped with the Mo (0.71073 Å) Kα radiation source, graphite monochromator, while for **8** were collected using the XtaLAB Synergy-S diffractometer, equipped with the Cu (1.54184 Å) Kα radiation source and graphite monochromator. Positions of all non-hydrogen atoms were determined by direct methods using the SIR-2014 [60] program. Refinement and further calculations were carried out using the SHELXL-2018 program [61]. All non-hydrogen atoms were refined anisotropically using weighted full-matrix least-squares on F^2^. The hydrogen atoms attached to oxygen atoms were identified on different Fourier maps, whereas all hydrogen atoms bonded to carbon atoms were included in the structure at idealized positions and were refined using a riding model with U_iso_ (H) fixed at 1.2 U_eq_ of C and 1.5 U_eq_ for methyl groups. In the crystal structures of **7** and **9**, a water molecule is present in the crystal lattice, wherein for **9** the occupancy factor of 0.3 of water molecule was used for refinement, and for molecular graphics, the MERCURY [62] program was used.

**7**: C_25_H_30_Cl_2_FO_3_N_4_^+^Cl^−^·H_2_O, Mr = 577.89, crystal size = 0.12 × 0.21 × 0.49 mm^3^, triclinic, space group P$\bar{1}$, a = 7.9131(3) Å, b = 13.0967(5) Å, c = 14.1077(5) Å, α = 68.230(3)°, β = 82.945(3)°, γ = 86.697(3)°, V = 1347.4(1) Å3, Z = 2, T = 130(2) K, 18,567 reflections collected, 6279 unique reflections (Rint = 0.0260), R1 = 0.0532, wR2 = 0.1416 [I > 2σ(I)] and R1 = 0.0683, wR2 = 0.1551 [all data].

**8**: C_25_H_30_Cl_2_FO_3_N_4_^+^Cl^−^, Mr = 559.88, crystal size = 0.07 × 0.17 × 0.30 mm^3^, triclinic, space group P$\bar{1}$, a = 7.8606(1) Å, b = 13.0752(2) Å, c = 14.4597(2) Å, α = 64.936(2)°, β = 81.780(1)°, γ = 88.492(1)°, V = 1331.4(4) Å3, Z = 2, T = 100(2) K, 36,993 reflections collected, 5449 unique reflections (Rint = 0.0439), R1 = 0.0401, wR2 = 0.1036 [I > 2σ(I)] and R1 = 0.0421, wR2 = 0.1052 [all data].

**9**: C_25_H_30_Cl_2_F_2_O_3_N_4_^+^Cl^−^·0.3H_2_O, Mr = 583.28, crystal size = 0.26 × 0.45 × 0.85 mm^3^, triclinic, space group P$\bar{1}$, a = 7.9118(3) Å, b = 13.0975(4) Å, c = 14.4313(5) Å, α = 65.905(2)°, β = 81.045(3)°, γ = 88.293(3)°, V = 1347.56(9) Å3, Z = 2, T = 130(2) K, 18,752 reflections collected, 6313 unique reflections (Rint = 0.0228), R1 = 0.0503, wR2 = 0.1250 [I > 2σ(I)] and R1 = 0.0630, wR2 = 0.1348 [all data].

CCDC 2248728-2248730 contains the supplementary crystallographic data. These data can be obtained free of charge from The Cambridge Crystallographic Data Centre via www.ccdc.cam.ac.uk/data_request/cif (accessed on 14 March 2023).

### 4.3. Pharmacology

#### 4.3.1. Chemicals

The following drugs and chemicals were used: thiopental (Rotexmedica, Trittau, Germany), heparin 5000 IU/mL (WZF Polfa S.A., Warsaw, Poland), ethyl alcohol 99.9% (Chempur, Piekary Śląskie, Poland), kolliphor (Sigma-Aldrich, Taufkirchen, Germany), sodium chloride (POCH, Gliwice, Poland), urapidil hydrochloride (Sigma-Aldrich, Germany), prazosin hydrochloride (Sigma-Aldrich, Germany), terazosin hydrochloride (Sigma-Aldrich, Germany), tamsulosin hydrochloride (Sigma-Aldrich, Germany), and phenylephrine (Sigma-Aldrich, Germany).

#### 4.3.2. Animals

The experiments were carried out on male normotensive Wistar rats (outbred stock, name: KRF:WI(WU)) weighing 250 to 300 g. The animals were purchased from a licensed breeder (Animal House, Faculty of Pharmacy, Jagiellonian University Medical College, Krakow, Poland). The rats were housed in pairs in standard plastic cages at a constant room temperature of 22–24 °C, relative humidity of 55 ± 10%, with a 12:12 h light/dark cycle (light on from 7 a.m. to 7 p.m.). The animals had free access to food pellets (Agropol S.J., Motycz, Poland) and filtered tap water. The experimental groups consisted of six animals each. The rats were adopted for 5 days to the laboratory conditions before being used in experiments. The animals were killed by cervical dislocation immediately after the experiments. All experimental procedures were performed according to the European Union Directive of 22 September 2010 (2010/63/EU) and Polish legislation concerning animal care and use and approved by the 2nd Local Ethics Committee for Experiments on Animals in Krakow, Poland (Resolution No. 127/2017).

#### 4.3.3. In Vitro Binding Assay—Determination of Affinity for α1-ARs

The affinity for α_1_-ARs was determined by radioligand binding assay on rat cerebral cortex using [3H]-prazosin as a specific radioligand. The brains were homogenized in 20 volumes of an ice-cold 50 mM Tris-HCl buffer (pH 7.6) and were centrifuged (MPW Med. Instruments, Warsaw, Poland) at 20,000× *g* for 20 min (0–4 °C). The cell pellet was resuspended in the Tris-HCl buffer and centrifuged again. Radioligand-binding assay was performed in plates (MultiScreen/Millipore, Burlington, MA, USA). The final incubation mixture (final volume 300 μL) consisted of 240 μL of the membrane suspension, 30 μL of [3H]-Prazosin (0.2 nM) solution, and 30 μL of the buffer containing seven to eight concentrations (10^−11^ to 10^−4^ M) of the tested compounds. For measuring the unspecific binding, phentolamine, 10 μM (in the case of [3H]-Prazosin) was applied. The incubation was terminated by rapid filtration over glass fiber filters (Whatman GF/C, Sigma-Aldrich) using a vacuum manifold (Millipore). The filters were then washed twice with the assay buffer and placed in scintillation vials with a liquid-scintillation cocktail. Radioactivity was measured in a WALLAC 1409 DSA liquid-scintillation counter (BioSurplus, San Diego, CA, USA).

#### 4.3.4. In Vitro Functional Assays—Determination of Intrinsic Activity at the α_1A_-ARs and the α_1B_-ARs

Intrinsic activity at the α_1_-AR subtypes studies were performed using the cells expressing mitochondrially-targeted aequorin and one of the human adrenergic receptor α_1A_ or α_1B_ (PerkinElmer, Boston, MA, USA), according to the manufacturer’s instructions. For measurement, frozen cells were thawed and re-suspended in 10 mL of assay buffer containing 5 μM “Coelenterazine h”. This cell suspension was put in a 10 mL Falcon tube, fixed onto a rotating heel, and incubated overnight at rt in the dark (8 rpm; 45° angle). Cells were diluted with Assay Buffer to 5000 cells/20 μL. Agonistic ligands 2 × (50 μL/well), diluted in Assay Buffer, were prepared in ½ white polystyrene area plates, and the cell suspension was dispensed in 50 μL volume on the ligands using the injector. The light emitted was recorded for 20 s. Cells with antagonists were incubated for 15 min at room temperature. Thereafter, 50 μL of the reference agonist phenylephrine (3 × EC_80_ final concentration) was injected into the mix of cells and antagonist, and the light emitted was recorded for 20 s. Luminescence was measured using the multidetection microplate reader (POLARstar Omega, BMG LABTECH, Ortenberg, Germany).

#### 4.3.5. Influence on Blood Pressure in Normotensive Rats

The normotensive rats were anesthetized with thiopental (75 mg/kg) by intraperitoneal injection (i.p.). The left carotid artery was cannulated with a polyethylene tub filled with heparin in saline to facilitate pressure measurements using a PowerLab Apparatus (ADInstruments, Sydney, Australia). Blood pressure was measured immediately before administration of the tested compounds or vehicle—time 0 min (initial blood pressure) and 60 min thereafter. Initial blood pressure before administration of the tested chemicals in all experimental groups was similar. The tested compounds were dissolved in a specific vehicle: ethyl alcohol 5% − kolliphor 5% − water 90%. All tested chemicals were administrated intravenously (i.v.) at a constant volume of 1 mL/kg after 20 min of stabilization period. The compounds were tested at a starting dose of 2 mg/kg. If the hypotensive activity was found, the dose was reduced by half. The dosing was continued until the hypotensive effects disappeared. Urapidil was used as a reference compound.

#### 4.3.6. Statistical Analysis

All statistics were performed using GraphPad Prism, Version 6.0 (GraphPad Software, San Diego, CA, USA). In the radioligand binding study, data were analyzed by a one-site curve-fitting equation, and inhibition constants (*K_i_*) values were calculated via the Cheng-Prusoff equation [63]. In functional studies, data were analyzed using a sigmoidal dose-response (variable slope) equation. Arterial blood pressure data were analyzed using a two-way ANOVA with Sidak’s post-hoc test for multiple comparisons. A *p* < 0.05 was considered statistically significant.

### 4.4. Molecular Modelling

All the compounds were prepared for docking using LigPrep [64] from the Schrödinger Suite using default settings. Docking was carried out in Glide [65], using extra precision mode. The compounds were docked to the homology models of α_1A_ and α_1B_, constructed on the basis of the GPCRdb data [66]. Homology modeling was carried out using the Modeller software version 9.13 [67] using a multi-template approach and available crystal structures of receptors from the aminergic family of GPCRs. The constructed models were evaluated by docking of known ligands and sets of inactive compounds towards α_1A_ and α_1B_. For each receptor subtype, one model providing the best discrimination between active and inactive compounds (in terms of the Area Under Receiver Operating Characteristic—AUROC) was selected. Visualizations of docking poses were prepared in Pymol [68].

## 5. Conclusions

This study reported the design, synthesis and pharmacological evaluation of a series of novel phenylpiperazine derivatives of 5,5-dimethylhydantoin as α_1_-AR antagonists. The newly synthesized compounds showed high-to-moderate affinity for α_1_-ARs. Compounds containing the methoxy group at position *ortho* of the phenylpiperazine phenyl ring revealed in vitro strong antagonistic activity at α_1A_- and/or α_1B_-AR and in vivo hypotensive effects. The most promising compound (**5**) exhibited a stronger antagonistic effect at α_1B_-ARs (IC_50_ = 0.002 nM) than classic α_1_-AR blockers such as prazosin, terazosin, tamsulosin and significantly decreased systolic and diastolic blood pressure after i.v. administration at a dose 0.0625 mg/kg. The hypotensive dose was lower than the effective dose obtained for the reference drug urapidil. Compound (**11**) possessing a 2-cyano- substituent at the phenyl ring of phenylpiperazine moiety demonstrated in vitro strong antagonistic activity at α_1A_-AR without significant changes in the hemodynamic parameters (SBP and DBP). Both compounds (**5** and **11**) are good candidates for further pharmacomodulation and biological screening development in search of innovative pharmacotherapy against circulation and urinary diseases. 

## Figures and Tables

**Table 1:**
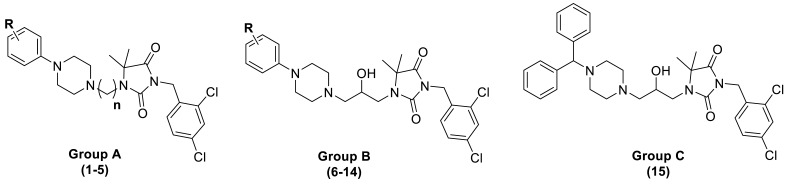
Structures of obtained 5,5-dimethylhydantoin derivatives (**1**–**15**).

Cpd	Group	n	R	Cpd	Group	n	R
**1**	A	3	2-MeO	**9**	B	-	2,4-diF
**2**	A	3	2,3-diCl	**10**	B	-	3,4-diCl
**3**	A	5	H	**11**	B	-	2-CN
**4**	A	6	3,4-diCl	**12**	B	-	3,4-diMe
**5**	A	6	2-MeO	**13**	B	-	2-MeO
**6**	B	-	H	**14**	B	-	3-MeO
**7**	B	-	2-F	**15**	C	-	-
**8**	B	-	4-F				

**Table 2:** α_1_-adrenoceptor affinity (*Ki*) for the tested compounds (**1**–**15**) and references (**AZ-99**, prazosin, and urapidil) [39,45].

Compound	*K_i_* (nM)
**1**	31.0
**2**	535.0
**3**	43.0
**4**	255.0
**5**	12.7
**6**	53.0
**7**	100.0
**8**	44.0
**9**	42.0
**10**	778.0
**11**	26.0
**12**	270.0
**13**	29.0
**14**	289.0
**15**	2443.0
**AZ-99**	529.0 ^a^
**Prazosin**	0.24 ^a^
**Urapidil**	127.9 ^b^

Inhibition constants (*K_i_*) values were obtained from three independent experiments in duplicates. ^a^ Results were published previously [45]; ^b^ Result was published previously [39].

**Table 3:** Intrinsic activity at the α_1_-ARs (α_1A_, α_1B_) for tested compounds and references.

Compound	α_1A_-AR		α_1B_-AR		Selectivity Ratio	
	IC_50_^a^ (nM)	Profile	IC_50_^a^ (nM)	Profile	α_1A_/α_1B_^b^	α_1B_/α_1A_^c^
**1**	90.62	Antagonist	10.71	Antagonist	8.46	0.12
**2**	1730.00	Antagonist	1146.00	Antagonist	1.51	0.66
**3**	290.00	Antagonist	3.27	Antagonist	88.73	0.01
**5**	0.02	Antagonist	0.002	Antagonist	10.00	0.10
**6**	5909.00	Antagonist	905.00	Antagonist	6.53	0.15
**7**	1415.00	Antagonist	483.90	Antagonist	2.92	0.34
**8**	816.70	Antagonist	170.40	Antagonist	4.79	0.21
**9**	162.40	Antagonist	147.40	Antagonist	1.10	0.91
**11**	162.30	Antagonist	359.10	Antagonist	0.45	2.21
**12**	3136.00	Antagonist	377.30	Antagonist	8.31	0.12
**13**	138.70	Antagonist	331.10	Antagonist	0.42	2.39
**14**	1221.00	Antagonist	1366.00	Antagonist	0.89	1.12
**Tamsulosin**	0.03	Antagonist	0.48	Antagonist	0.06	16.00
**Terazosin**	4.52	Antagonist	0.67	Antagonist	6.70	0.15
**Prazosin**	0.72	Antagonist	0.17	Antagonist	4.25	0.24
**Phenylephrine**	23.64	Agonist	2.31	Agonist	10.25	0.10

^a^ IC_50_ values are means of three independent experiments in duplicates; ^b^ selectivity for α_1B_-AR (IC_50(α1A-AR)_/IC_50(α1B-AR)_); ^c^ selectivity for α_1A_-AR (IC_50(α1B-AR)_/IC_50(α1A-AR)_).

## Data Availability

Data supporting reported results can be found in the Supplementary file provided during submission online.

## Supplementary Materials

The following supporting information can be downloaded at: https://www.mdpi.com/article/10.3390/ijms242316609/s1.
