# Supplementary material for: The Subtype Selectivity in Search of Potent Hypotensive Agents among 5,5-Dimethylhydantoin Derived α1-Adrenoceptors Antagonists
Source: Int J Mol Sci. 2023 Nov 22;24(23):16609. doi: 10.3390/ijms242316609 (PMC10706087; doi:10.3390/ijms242316609)
Supplement: Supplementary file 1 [file ijms-24-16609-s001.zip › ijms-2715366-supplementary.pdf]

Supplementary materials

The subtype selectivity in search of potent hypotensive agents among 5,5-dimethylhydantoin derived α1-adrenoceptors antagonists

Aneta Kaczor [1†], Joanna Knutelska [2†], Katarzyna Kucwaj-Brysz [1], Małgorzata Zygmunt [2], Ewa Żesławska [3], Agata Siwek [4], Marek Bednarski [2], Sabina Podlewska [5], Magdalena Jastrzębska-Więsek [6], Wojciech Nitek [7], Jacek Sapa [2] and Jadwiga Handzlik [1,*]

^{1}HNMRs for final compounds……..……………………………………………………..2-13

The synthetic procedure for intermediate 17……………………………..14

The influence on blood pressure in rats………………………………15-16

^{1}HNMRs

for final products obtained within the studies

Compound 1

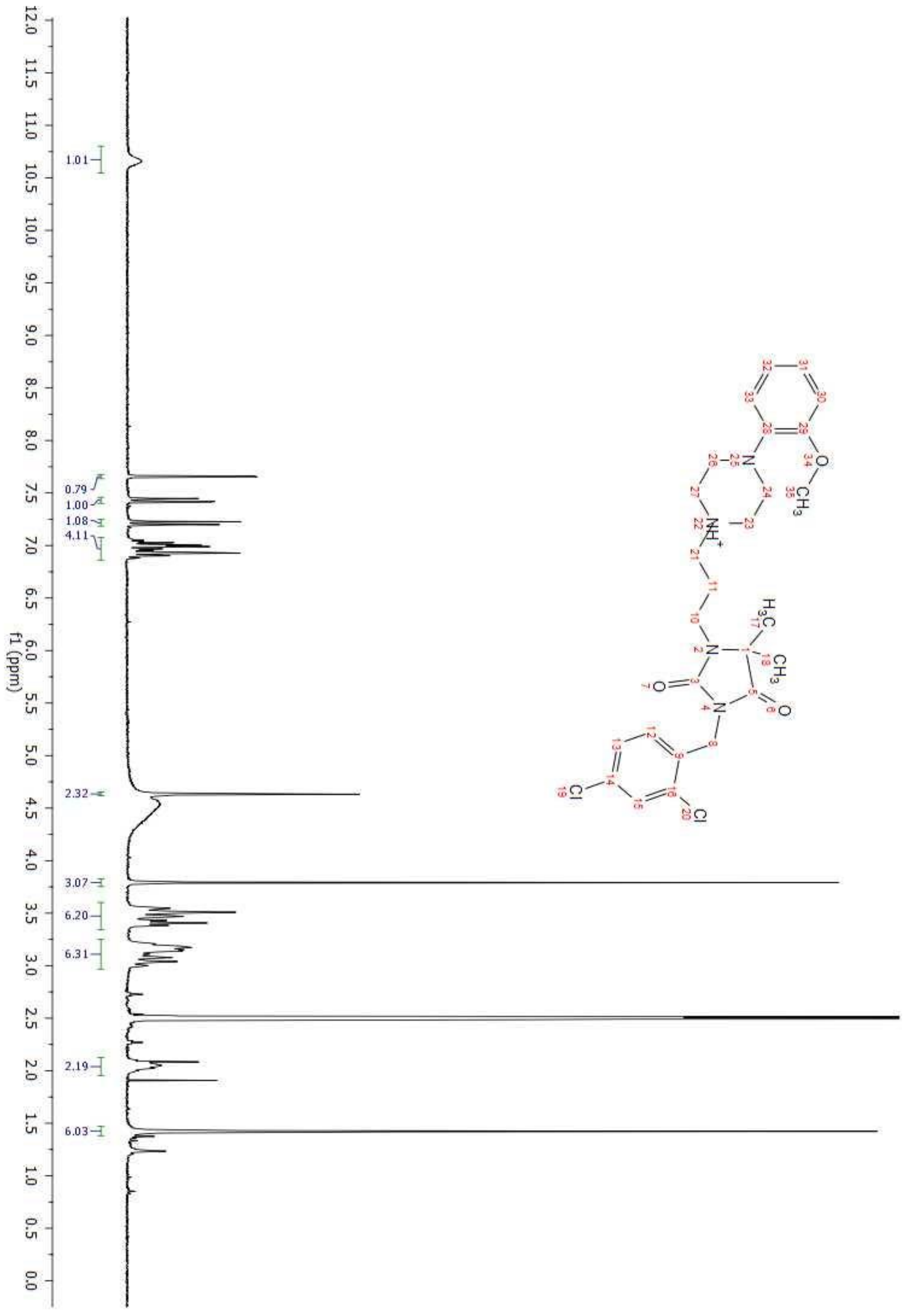

Compound 2

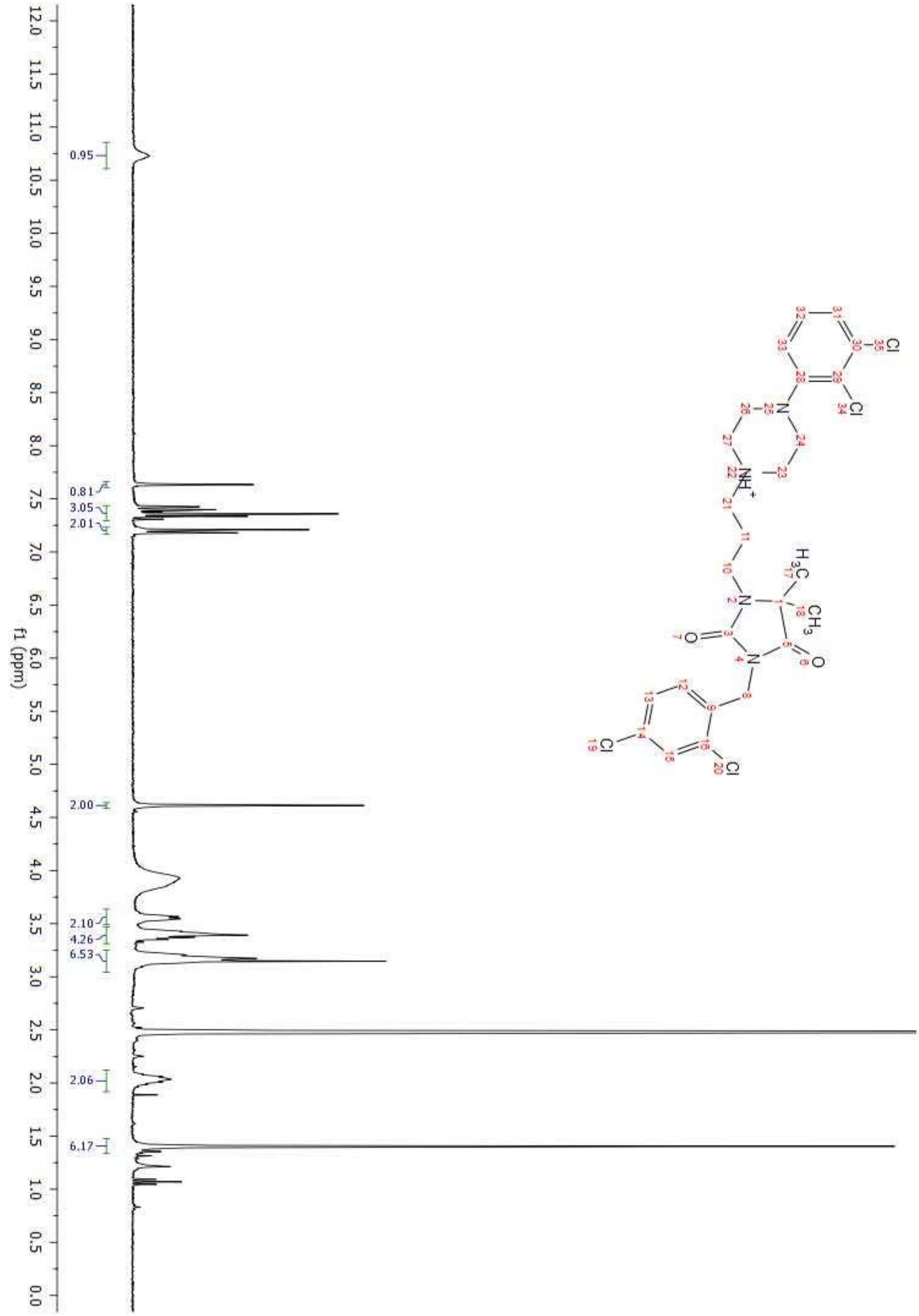
f1 (ppm)
12.0
11.5
11.0
10.5
10.0
9.5
9.0
8.5
8.0
7.5
7.0
6.5
6.0
5.5
5.0
4.5
4.0
3.5
3.0
2.5
2.0
1.5
1.0
0.5
0.0
0.95
0.81
3.05
2.01
2.00
2.10
4.26
6.53
2.06
6.17
Cl
Cl
NH+
H3C
CH3
O
O
Cl
Cl

Compound 3

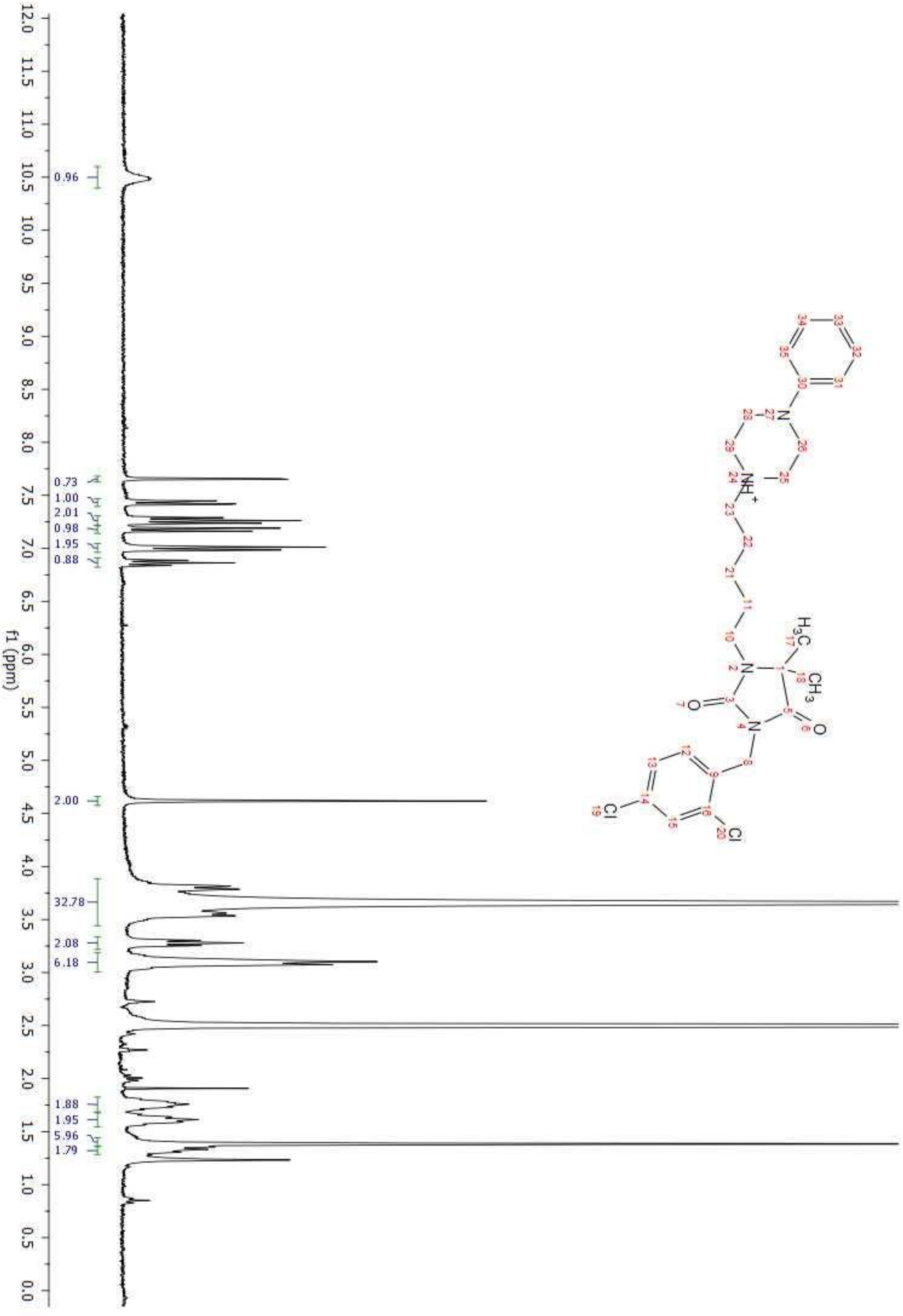

f1 (ppm)
0.96
0.73
1.00
2.01
0.98
1.95
0.88
2.00
32.78
2.08
6.18
1.88
1.95
5.96
1.79
NH+
H3C
CH3
O
O
N
N
Cl
Cl

Compound 4

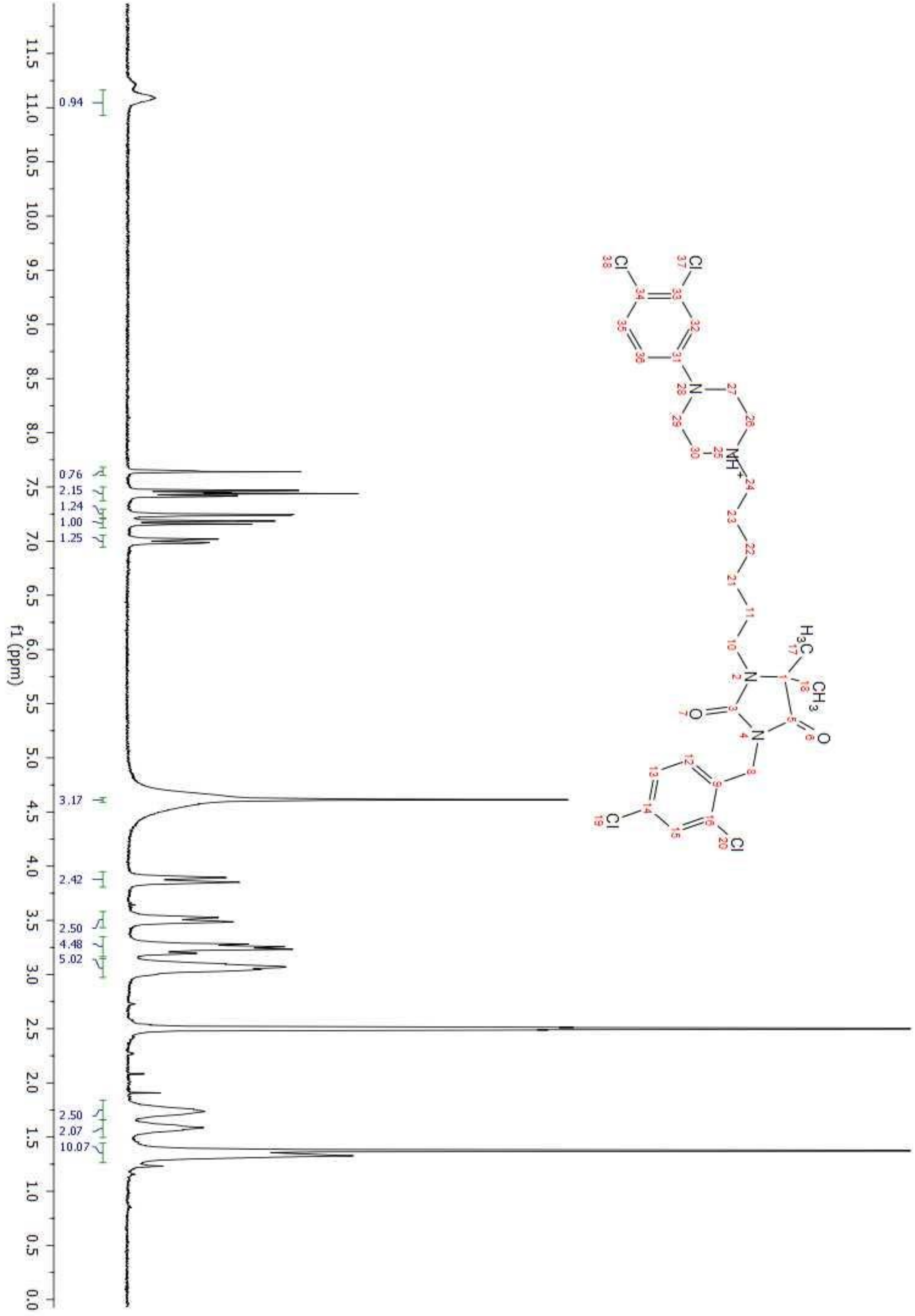

Compound 5

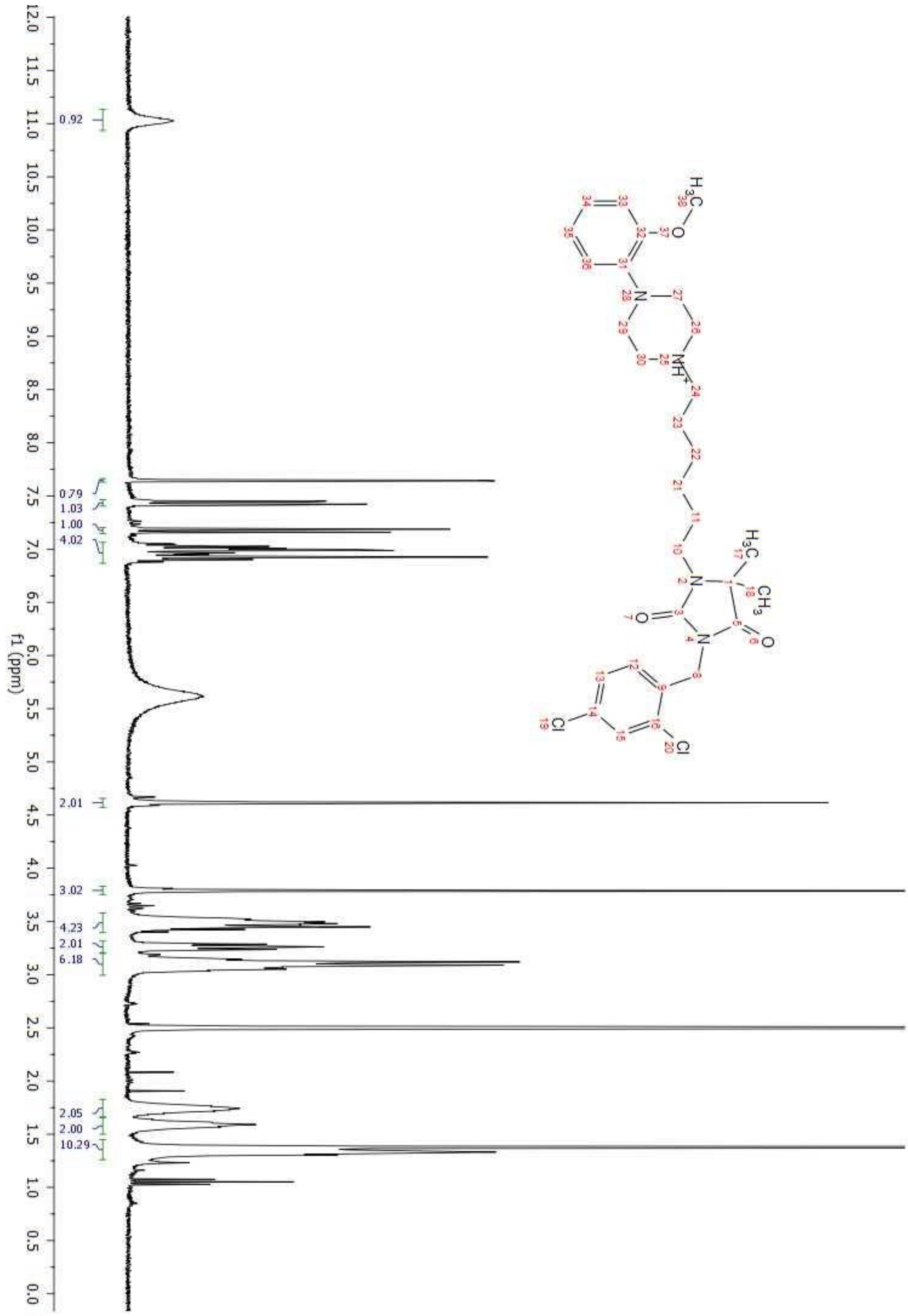

Compound 6

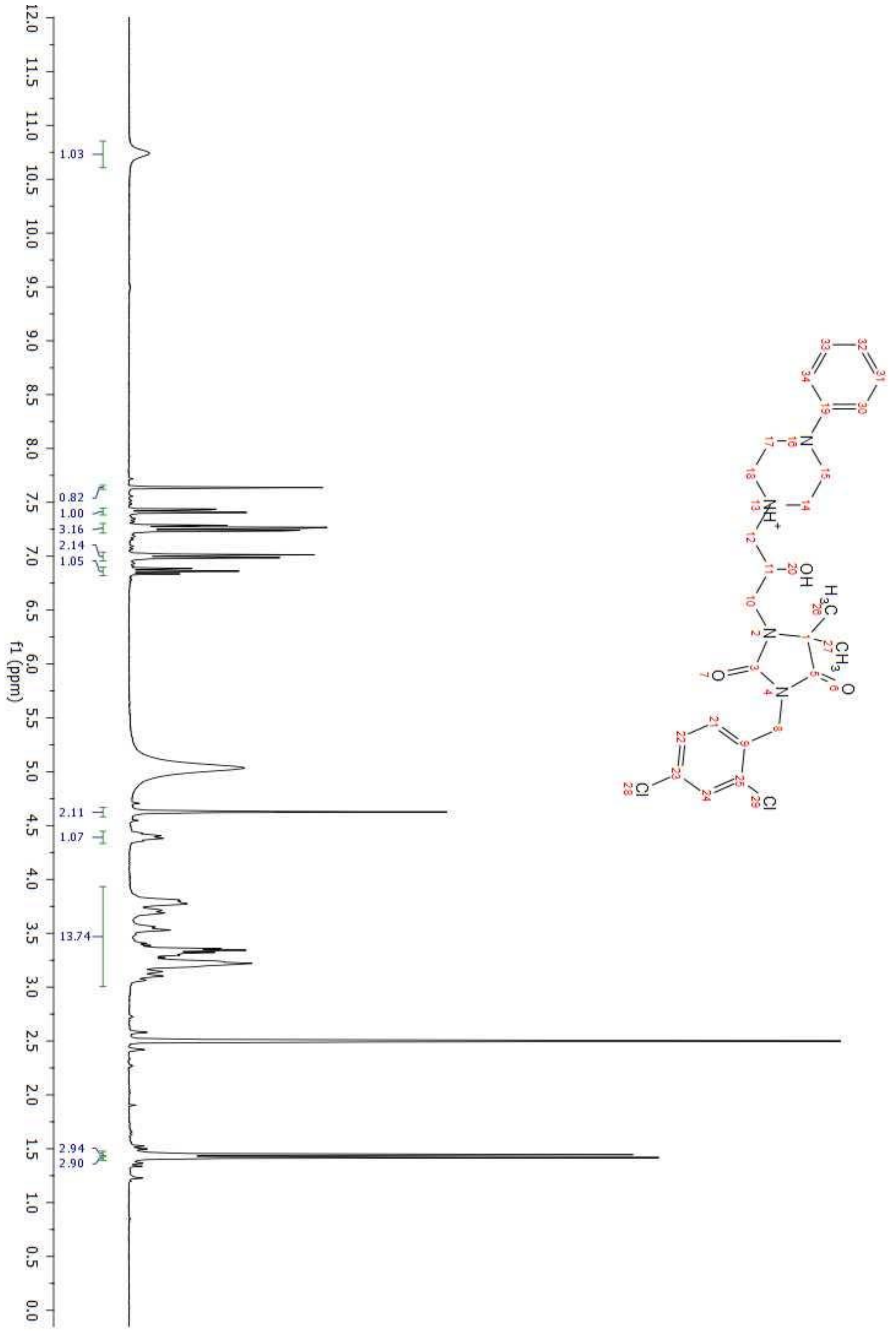

f1 (ppm)
1.03
0.82
1.00
3.16
2.14
1.05
2.11
1.07
13.74
2.94
2.90
OH
H_3C
CH_3
NH^+
N
O
Cl

Compound 10

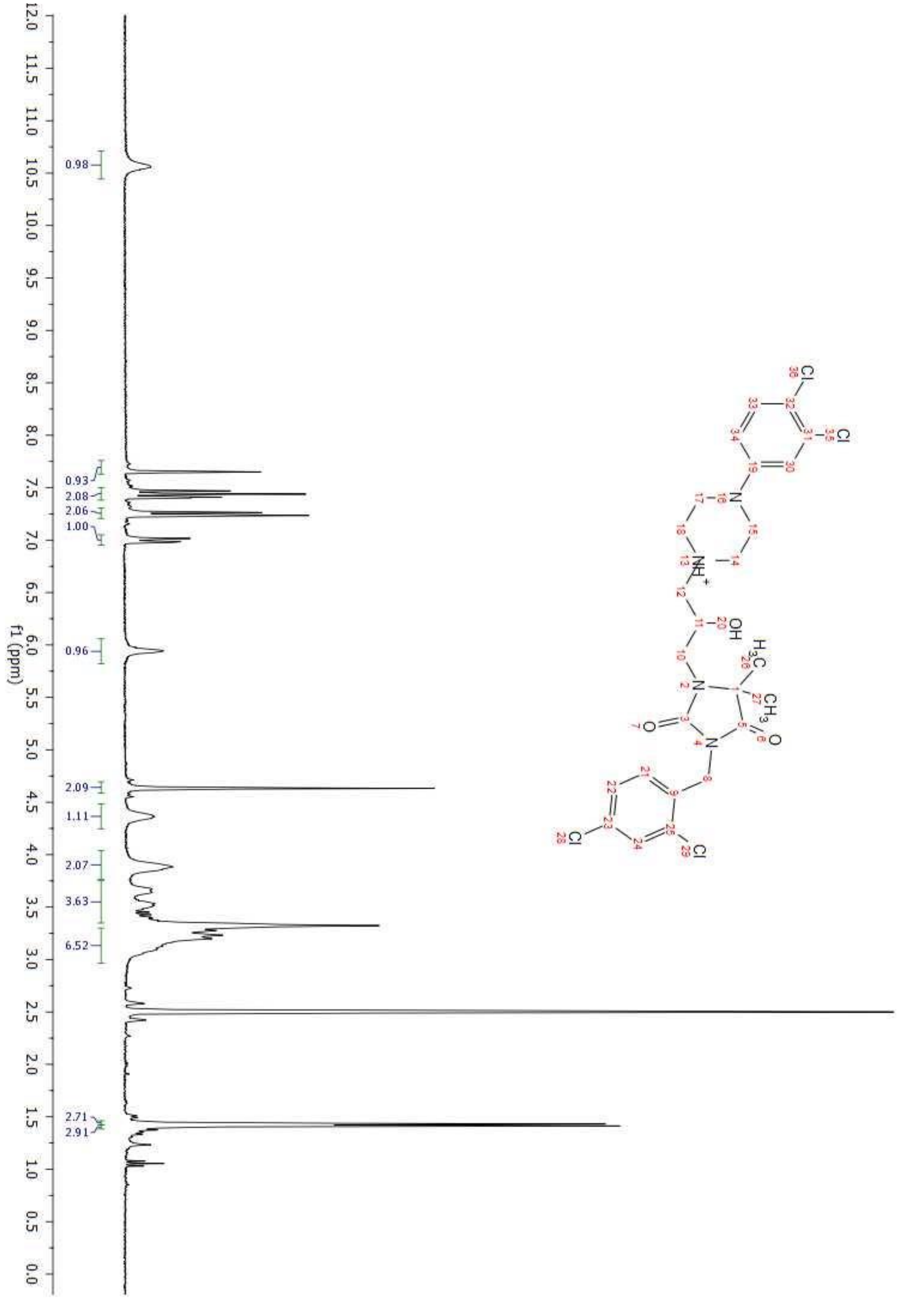

f1 (ppm)
0.98
0.93
2.08
2.06
1.00
0.96
2.09
1.11
2.07
3.63
6.52
2.71
2.91
OH
CH3
H3C
O
Cl
Cl
Cl
Cl
NH+

Compound 11

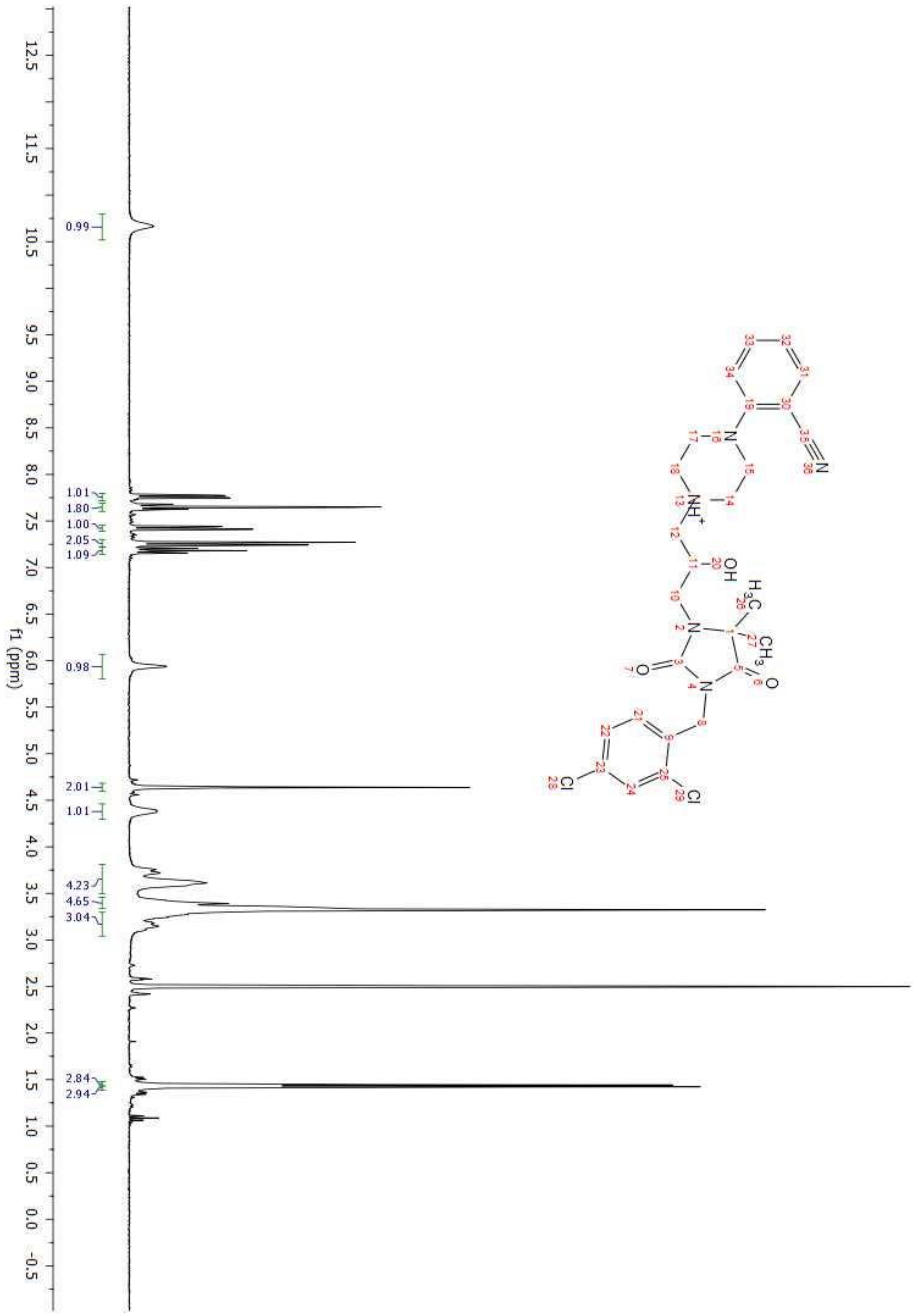

f1 (ppm)
12.5
11.5
10.5
9.5
9.0
8.5
8.0
7.5
7.0
6.5
6.0
5.5
5.0
4.5
4.0
3.5
3.0
2.5
2.0
1.5
1.0
0.5
0.0
-0.5
0.99
1.01
1.80
1.00
2.05
1.09
0.98
2.01
1.01
4.23
4.65
3.04
2.84
2.94
NH+
OH
H3C
CH3
O
N
Cl

Compound 12

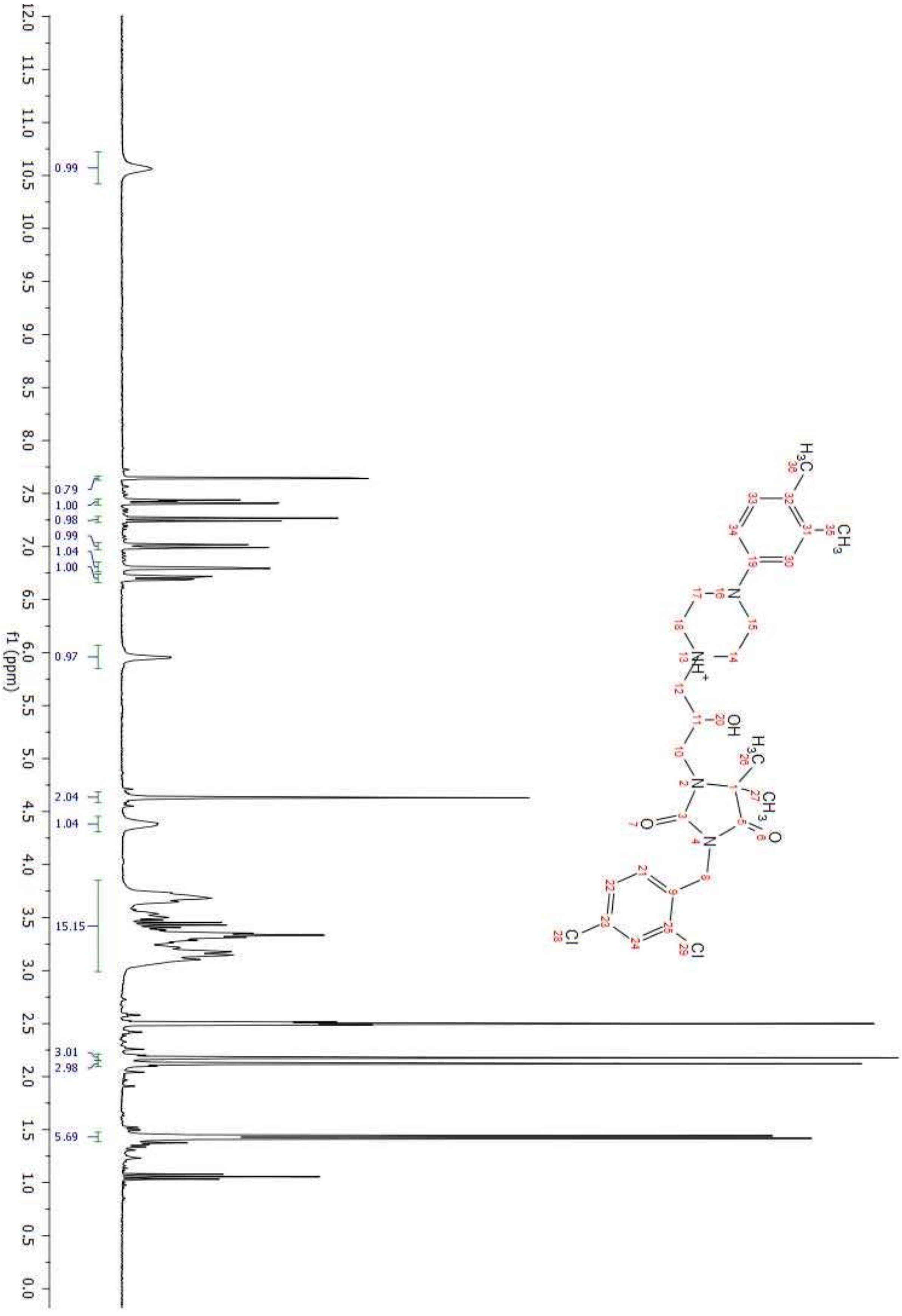

Compound 13

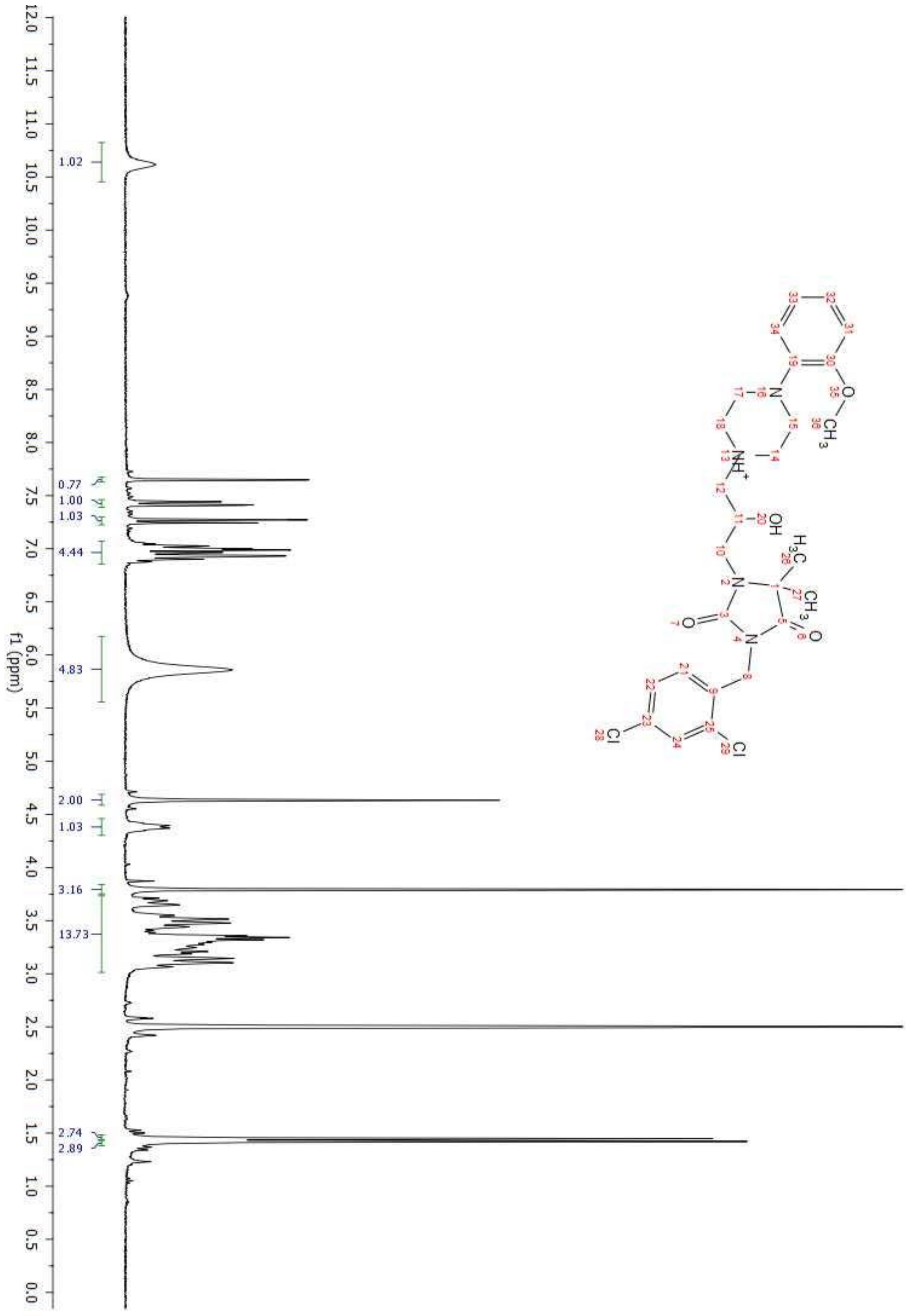

Compound 14

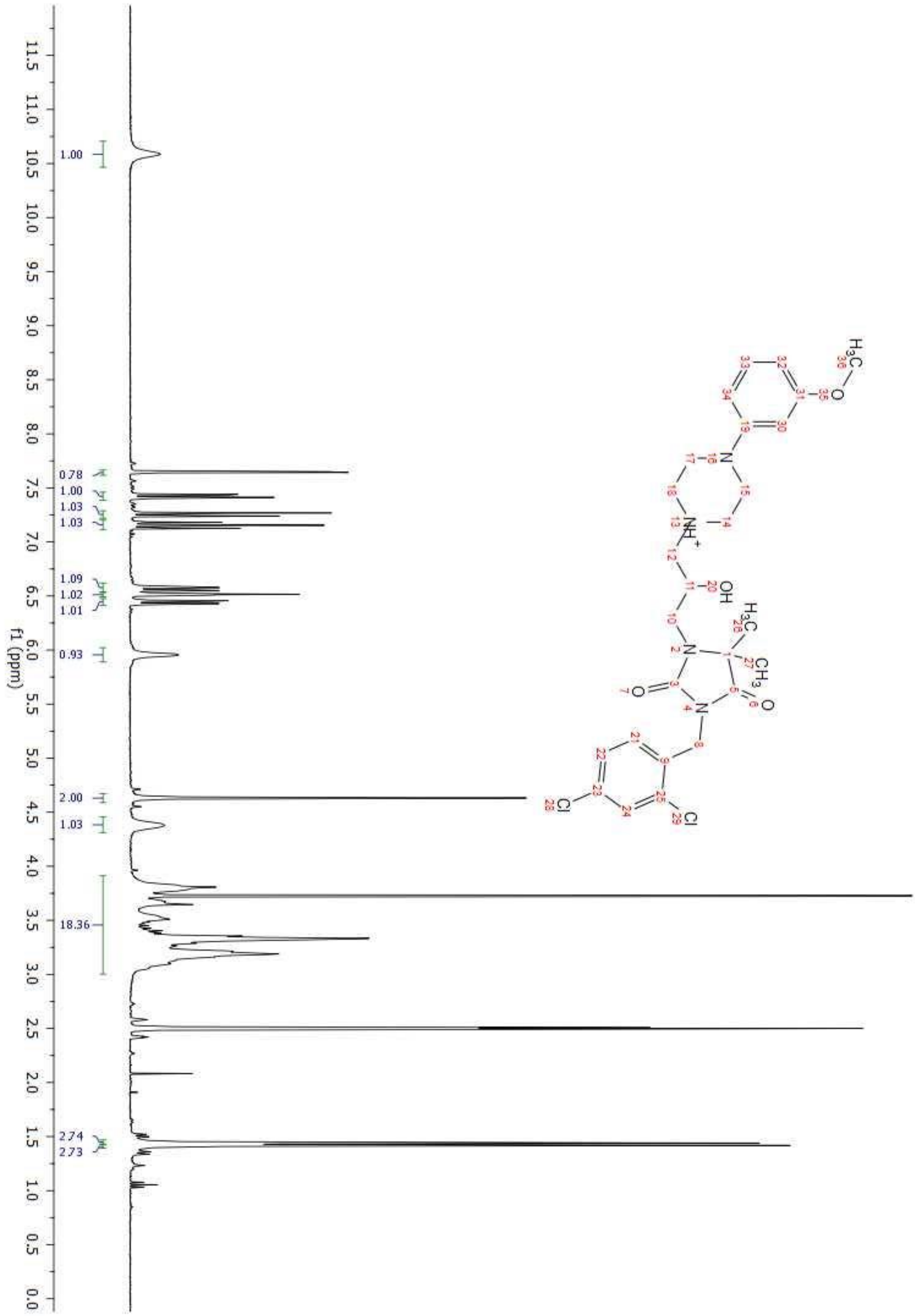

The synthetic procedure for intermediate 17

3-(2,4-dichlorobenzyl)-5,5-dimethylimidazolidine-2,4-dione (**16**) (40 mmol, 11.48 g), potassium carbonate (11.58 mmol, 16.0 g), TEBA (5.27 mmol, 1.2 g) and acetone (80 ml) was stirred for 30 min. Then, 1,3-dibromopropan (60 mmol, 12.11 g) in acetone (40 ml) was added. It was stirred for 4 days. Filtration was carried out and filtrate was evaporated. To the residue, dichloromethane was added and mixture was washed three times with 1% sodium hydroxide and twice with water. Organic fractions were dried, then filtrated and evaporated. Crude product was purified from reaction mixture using column chromatography in dichloromethane:acetone (10:1). As these reaction has been well established in our team using analogous starting materials [1,2], the resulted white solid, pure according to TLC control was directly used for synthesis of final compounds.

1. Handzlik, J.; Bojarski, A. J.; Satała, G.; Kubacka, M.; Sadek, B.; Ashoor, A.; Siwek, A.; Więcek, M.; Kucwaj, K.; Filipek, B.; Kieć-Kononowicz, K. SAR-studies on the importance of aromatic ring topologies in search for selective 5-HT(7) receptor ligands among phenylpiperazine hydantoin derivatives. *Eur J Med Chem.* **2014**, *78*, 324–339. doi: 10.1016/j.ejmech.2014.01.065.
2. Kononowicz, K.; Handzlik, J. Phenylpiperazine 5,5-Dimethylhydantoin Derivatives as First Synthetic Inhibitors of Msr(A) Efflux Pump in Staphylococcus epidermidis. *Molecules.* **2020**, *25*, 3788. doi: 10.3390/molecules25173788.

The influence on blood pressure in rats

Table S1. Influence on blood pressure for vehicle, tested compounds (**1**-**3**, **5**-**9**, **11**-**14**), and urapidil.

Cpd	Dose (mg/kg)	Blood pressure	Time of observation (min)							
			0	5	10	20	30	40	50	60
Vehicle	–	Systolic	133.2 ± 3.5	133.8 ± 3.1	130.3 ± 3.0	131.0 ± 3.2	130.3 ± 3.0	129.7 ± 3.5	132.2 ± 4.1	132.3 ± 3.5
		Diastolic	98.3 ± 3.3	98.0 ± 2.9	94.8 ± 2.5	95.7 ± 3.2	95.2 ± 3.0	95.2 ± 2.9	96.0 ± 3.6	96.3 ± 3.0
**1**	2.0	Systolic	133.3 ± 4.5	120.3 ± 3.1	113.0 ± 3.1**	112.2 ± 3.6**	114.2 ± 3.7*	114.7 ± 3.9*	116.0 ± 3.8*	117.5 ± 4.3*
		Diastolic	101.0 ± 3.1	88.3 ± 2.3	82.3 ± 3.3*	82.2 ± 4.2*	81.3 ± 3.9*	80.8 ± 3.1*	81.7 ± 2.3*	83.3 ± 2.1*
**2**	2.0	Systolic	129.3 ± 5.8	126.7 ± 5.6	124.2 ± 5.3	121.7 ± 6.3	121.2 ± 5.6	120.2 ± 5.7	120.3 ± 4.7	119.3 ± 5.1
		Diastolic	100.7 ± 4.3	98.5 ± 4.4	96.8 ± 4.0	92.8 ± 3.8	90.3 ± 3.9	89.3 ± 3.6	88.0 ± 3.1	88.0 ± 2.7
**3**	2.0	Systolic	132.2 ± 1.6	123.3 ± 1.7	113.7 ± 1.3***	113.0 ± 2.2***	116.3 ± 2.2**	116.3 ± 1.9**	120.3 ± 1.7*	119.7 ± 2.4*
		Diastolic	93.7 ± 2.5	90.3 ± 2.0	81.2 ± 2.3**	81.7 ± 2.6**	83.2 ± 2.4*	82.7 ± 2.1*	84.2 ± 2.6*	83.8 ± 3.3*
**5**	0.0625	Systolic	128.8 ± 2.0	121.0 ± 3.0*	116.8 ± 2.6**	114.2 ± 2.3***	113.3 ± 2.0***	114.7 ± 1.4**	116.8 ± 0.6**	116.3 ± 1.2***
		Diastolic	92.0 ± 1.9	87.7 ± 1.6*	84.2 ± 1.6*	81.7 ± 1.8**	82.7 ± 1.5**	81.7 ± 2.2**	81.8 ± 1.6**	83.3 ± 2.3**
**6**	2.0	Systolic	134.2 ± 2.8	124.2 ± 4.2	125.2 ± 3.7	126.3 ± 3.6	125.0 ± 4.0	126.7 ± 3.9	125.5 ± 4.4	125.8 ± 3.6
		Diastolic	98.2 ± 3.5	87.2 ± 4.8	90.3 ± 4.7	89.7 ± 4.4	89.0 ± 4.7	89.0 ± 4.5	89.2 ± 5.1	88.7 ± 4.6
**7**	2.0	Systolic	137.8 ± 5.5	125.3 ± 5.7	124.3 ± 5.9	121.8 ± 5.6	123.8 ± 6.0	123.7 ± 5.7	122.7 ± 6.0	121.5 ± 6.5
		Diastolic	103.2 ± 2.5	91.5 ± 4.2	91.0 ± 4.4	93.0 ± 4.6	92.0 ± 3.9	94.3 ± 4.7	93.2 ± 5.2	91.3 ± 4.2
**8**	2.0	Systolic	129.2 ± 6.9	121.5 ± 5.7	120.5 ± 6.3	120.0 ± 6.7	120.2 ± 6.7	119.5 ± 6.1	120.0 ± 6.5	119.0 ± 6.1
		Diastolic	96.7 ± 5.9	87.8 ± 6.5	89.0 ± 6.6	88.2 ± 5.9	86.5 ± 5.5	84.7 ± 5.2	85.5 ± 4.2	84.3 ± 4.6
**9**	2.0	Systolic	135.2 ± 8.0	133.3 ± 5.5	127.0 ± 6.5	125.0 ± 7.2	128.2 ± 7.0	128.2 ± 5.5	127.2 ± 5.0	122.0 ± 5.3
		Diastolic	100.3 ± 6.1	98.2 ± 4.4	93.5 ± 3.3	90.8 ± 4.0	93.2 ± 5.0	92.0 ± 4.9	90.3 ± 5.1	87.3 ± 3.8
**11**	2.0	Systolic	128.5 ± 5.2	117.0 ± 3.8	112.8 ± 5.1	113.0 ± 5.6	112.5 ± 5.9	113.8 ± 5.8	116.3 ± 6.0	118.2 ± 6.4
		Diastolic	91.7 ± 4.0	84.0 ± 4.4	80.7 ± 5.3	79.7 ± 5.6	79.8 ± 5.9	82.0 ± 5.9	82.3 ± 5.7	81.7 ± 6.1
**12**	2.0	Systolic	139.0	134.7	134.5	132.0	131.0	129.0	130.0	128. 0

			± 2.3	± 2.4	± 2.2	± 3.1	± 3.3	± 3.7	± 3.9	± 3.7
		Diastolic	104.2 ± 1.2	98.2 ± 1.9	99.5 ± 2.1	97.8 ± 1.8	96.7 ± 2.1	97.0 ± 2.0	96.8 ± 2.4	97.3 ± 2.0
**13**	2.0	Systolic	130.7 ± 4.6	102.8 ± 3.4****	107.7 ± 2.5****	109.3 ± 2.7***	112.7 ± 3.8**	113.3 ± 3.0*	117.2 ± 4.5*	118.0 ± 3.3*
		Diastolic	98.0 ± 3.2	61.2 ± 2.5****	70.8 ± 1.9****	77.3 ± 2.5***	82.2 ± 3.3*	82.8 ± 3.0*	82.2 ± 3.2*	83.7 ± 3.2*
**14**	2.0	Systolic	132.5 ± 4.0	132.3 ± 3.9	132.0 ± 4.2	129.3 ± 4.4	127.7 ± 3.8	125.8 ± 4.6	124.5 ± 4.5	124.3 ± 4.1
		Diastolic	100.8 ± 4.6	100.3 ± 4.3	100.7 ± 4.6	96.0 ± 4.0	96.0 ± 5.1	97.3 ± 4.7	94.3 ± 4.7	92.0 ± 4.7
**Urapidil**	1.0	Systolic	127.3 ± 2.9	111.3 ± 3.6***	109.5 ± 3.9***	110.2 ± 4.7***	112.2 ± 3.7**	112.7 ± 3.2**	114.0 ± 3.2**	115.0 ± 2.4**
		Diastolic	93.0 ± 2.7	77.5 ± 4.9***	75.5 ± 3.9***	76.0 ± 3.8***	78.8 ± 3.5**	79.2 ± 2.9**	80.8 ± 2.8*	81.2 ± 2.3*

Data represent the mean ± SEM (n = 6 rats per each group). Statistical analysis: two-way ANOVA and Sidak's multiple comparisons test. Statistically significant: *p < 0.05, **p < 0.02, ***p < 0.01, *****p< 0.001 vs. control group (vehicle treatment).
